# Prostate cancer resistance leads to a global deregulation of translation factors and unconventional translation

**DOI:** 10.1093/narcan/zcac034

**Published:** 2022-11-04

**Authors:** Emeline I J Lelong, Gabriel Khelifi, Pauline Adjibade, France-Hélène Joncas, Valérie Grenier St-Sauveur, Virginie Paquette, Typhaine Gris, Amina Zoubeidi, Etienne Audet-Walsh, Jean-Philippe Lambert, Paul Toren, Rachid Mazroui, Samer M I Hussein

**Affiliations:** Cancer Research Center, Université Laval, Quebec City, Québec G1R 3S3, Canada; CHU of Québec-Université Laval Research Center, Oncology Division, Quebec City, Québec G1R 3S3, Canada; Cancer Research Center, Université Laval, Quebec City, Québec G1R 3S3, Canada; CHU of Québec-Université Laval Research Center, Oncology Division, Quebec City, Québec G1R 3S3, Canada; Cancer Research Center, Université Laval, Quebec City, Québec G1R 3S3, Canada; CHU of Québec-Université Laval Research Center, Oncology Division, Quebec City, Québec G1R 3S3, Canada; Cancer Research Center, Université Laval, Quebec City, Québec G1R 3S3, Canada; CHU of Québec-Université Laval Research Center, Oncology Division, Quebec City, Québec G1R 3S3, Canada; Cancer Research Center, Université Laval, Quebec City, Québec G1R 3S3, Canada; CHU of Québec-Université Laval Research Center, Oncology Division, Quebec City, Québec G1R 3S3, Canada; Cancer Research Center, Université Laval, Quebec City, Québec G1R 3S3, Canada; CHU of Québec-Université Laval Research Center, Endocrinology and Nephrology Division, Quebec City, Québec G1V 4G2, Canada; Cancer Research Center, Université Laval, Quebec City, Québec G1R 3S3, Canada; CHU of Québec-Université Laval Research Center, Oncology Division, Quebec City, Québec G1R 3S3, Canada; Vancouver Prostate Centre, Department of Urologic Sciences, Faculty of Medicine, University of British Columbia, Vancouver, British Columbia V6H 3Z6, Canada; Cancer Research Center, Université Laval, Quebec City, Québec G1R 3S3, Canada; CHU of Québec-Université Laval Research Center, Endocrinology and Nephrology Division, Quebec City, Québec G1V 4G2, Canada; Cancer Research Center, Université Laval, Quebec City, Québec G1R 3S3, Canada; CHU of Québec-Université Laval Research Center, Endocrinology and Nephrology Division, Quebec City, Québec G1V 4G2, Canada; Cancer Research Center, Université Laval, Quebec City, Québec G1R 3S3, Canada; CHU of Québec-Université Laval Research Center, Oncology Division, Quebec City, Québec G1R 3S3, Canada; Cancer Research Center, Université Laval, Quebec City, Québec G1R 3S3, Canada; CHU of Québec-Université Laval Research Center, Oncology Division, Quebec City, Québec G1R 3S3, Canada; Cancer Research Center, Université Laval, Quebec City, Québec G1R 3S3, Canada; CHU of Québec-Université Laval Research Center, Oncology Division, Quebec City, Québec G1R 3S3, Canada

## Abstract

Emerging evidence associates translation factors and regulators to tumorigenesis. However, our understanding of translational changes in cancer resistance is still limited. Here, we generated an enzalutamide-resistant prostate cancer (PCa) model, which recapitulated key features of clinical enzalutamide-resistant PCa. Using this model and poly(ribo)some profiling, we investigated global translation changes that occur during acquisition of PCa resistance. We found that enzalutamide-resistant cells exhibit an overall decrease in mRNA translation with a specific deregulation in the abundance of proteins involved in mitochondrial processes and in translational regulation. However, several mRNAs escape this translational downregulation and are nonetheless bound to heavy polysomes in enzalutamide-resistant cells suggesting active translation. Moreover, expressing these corresponding genes in enzalutamide-sensitive cells promotes resistance to enzalutamide treatment. We also found increased association of long non-coding RNAs (lncRNAs) with heavy polysomes in enzalutamide-resistant cells, suggesting that some lncRNAs are actively translated during enzalutamide resistance. Consistent with these findings, expressing the predicted coding sequences of known lncRNAs *JPX, CRNDE* and *LINC00467* in enzalutamide-sensitive cells drove resistance to enzalutamide. Taken together, this suggests that aberrant translation of specific mRNAs and lncRNAs is a strong indicator of PCa enzalutamide resistance, which points towards novel therapeutic avenues that may target enzalutamide-resistant PCa.

## INTRODUCTION

Translation is one of the last processes in the flow of genetic information. It is a multistep and highly controlled protein synthesis process consisting of three major steps, namely initiation, elongation and termination (1). Translation initiation depends on a network of interacting translation initiation factors (eIFs) which are highly regulated. In particular, regulation of the activity and expression of eIFs, for example eIF4F or eIF2α, is under extensive study, and has revealed an important role for translation regulation in cellular processes such as cell differentiation, growth and cell stress response (2,3). Dysregulation of eIFs and altered expression or activity of components of the eIF4F complex such as eIF4E and eIF4G, have been observed to support cancer cell growth by activating translation initiation of mRNAs encoding key cell cycle regulators, as well as survival and oncogenic factors (4). Furthermore, eIF4E phosphorylation promotes prostate tumorigenesis and is elevated in prostate cancer (PCa), most notably in the castrate resistant form of the disease. This correlates with disease progression and poor clinical outcomes in patients with PCa (5). On the other hand, while phosphorylation of eIF2α blocks general translation in cases of cellular stress, it allows the preferential translation of a specific set of target mRNAs involved in cell adaptation to stress and survival (6). This is in line with recent evidence associating alterations in the phosphorylated eIF2α translational pathway with cancer, a process highly linked to the cellular stress response (7). Moreover, altered phosphorylation of eIF2α has been observed to occur as an adaptive stress response in both murine and humanized models of aggressive and resistant PCa (8). Perturbations in translation regulation may therefore represent key indicators of PCa severity.

PCa resistance is a highly prevalent and common cause of cancer-related death worldwide (9,10). Despite effective local treatments, many patients experience recurrences and eventually develop metastases (11–13). Highly dependent on androgens for growth, recurrent or metastatic PCa is treated with androgen deprivation therapy (ADT). Concomitant or subsequent use of enzalutamide (ENZ), a potent androgen receptor (AR) antagonist, significantly delays the consequences of treatment failure (14–16). However, not all patients benefit from the therapeutic effects of ENZ, and all eventually develop resistance (17). This highlights an urgent need to find reliable markers that can predict patient response and development of resistance. Recent genomics studies have addressed some of these issues and led to the discovery of promising PCa biomarkers (18–20). Due to the relative ease of nucleic acid sequencing, a large majority of existing PCa-related data focuses on transcriptomic studies analysing total RNA abundance as a stand-in for protein levels. This precludes discovery of many potential biomarkers whose protein expression relies mainly on the translational rate. Indeed, it is now well established that transcriptomic estimates of RNA abundance alone are insufficient to capture proteins whose differential expression critically impacts cellular differentiation and growth, environmental and pathological stress, or tumorigenesis (21,22). This is in part due to the complex regulatory mechanisms that orchestrate the translation of RNAs. It is estimated that about 40% of protein level variations are due to translational regulation (23). Thus, accurate estimation and identification of relevant protein variations occurring in various cancers including PCa calls for integrative methods that measure the transcriptome, the RNAs associated with translating polysomes (translatome), as well as the proteome.

Monitoring the translational status of entire transcripts via polysome profiling and RNA sequencing (RNA-seq) is a powerful approach used to identify ribosome-associated RNAs (24). Indeed, several polysome profiling studies on cancer cell lines have succeeded in identifying cancer cell-specific signatures not detected by standard RNA-seq analyses (25–27). Hsieh *et al.* reported the first study in PCa using polysome profiling (28). They found that eIF4F, driven by its upstream mTORC1 signaling regulatory pathway, promotes a metastatic phenotype in PCa through preferential translation of mRNAs encoding proteins involved in cell invasion and metastases. This is consistent with data revealing the PI3K/AKT/mTOR translational pathway as a key oncogenic pathway in treatment resistant PCa (29). Even though accumulating evidence supports a potential role played by translation regulation in the progression of PCa, the role of translational changes in the acquisition of ADT resistance or ENZ-resistance in PCa remains unknown.

To investigate perturbations in the translatome acquired upon PCa ENZ-resistance, we utilize an integrative approach merging global quantification of RNA abundance by RNA-seq and of their association to ribosomes through polysome profiling in ENZ-sensitive and ENZ-resistant PCa cell lines. We supplement these results with mass spectrometry of total proteins, patient proteomic data analyses and evaluation of phosphorylation states for core translation factors. We apply these methods to a novel model of castration-resistant (ENZ-sensitive) and ENZ-resistant PCa which we developed from the well-known AR-positive VCaP prostate cancer cell line (30). Some of these analyses were also validated in the ENZ-sensitive LNCaP and ENZ-resistant MR49F cell lines (31). We show that for a majority of mRNAs, translation is downregulated in ENZ resistant cells, but that a subset of mRNAs escapes this downregulation. We identify such mRNA candidates for which overexpression in ENZ-sensitive cells promotes development of resistance. Furthermore, our analysis revealed enrichment of long non-coding RNAs (lncRNAs) associated to ribosomes, which may suggest aberrant translation of novel peptides in the context of ENZ-resistant PCa. Again, overexpression of putative open reading frames (ORFs) of select lncRNAs in sensitive cells induces resistance to enzalutamide. Our findings thus point towards novel potential biomarkers or therapeutic targets which are involved in PCa resistance to ENZ.

## MATERIALS AND METHODS

### Generation of the enzalutamide-resistant VCaP^ER^ model in mice

**Figure 1.**
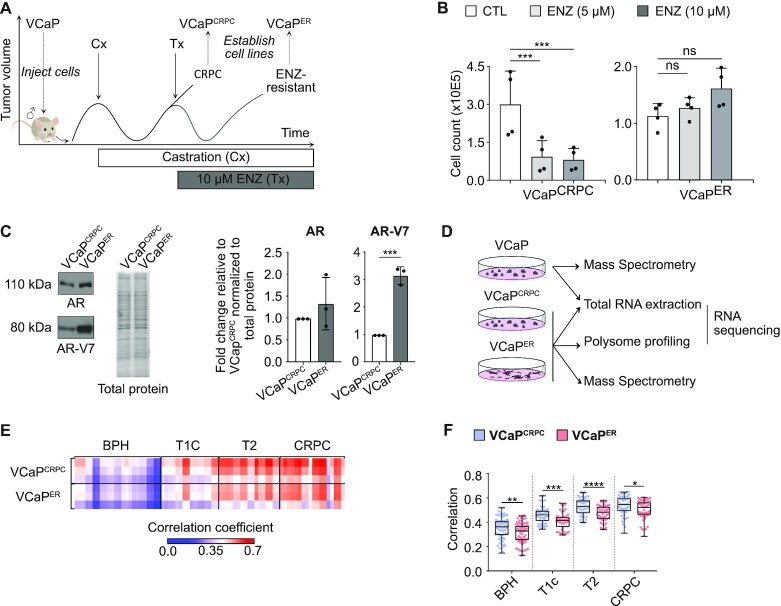
Establishment of enzalutamide (ENZ)-resistant prostate cancer (PCa) cellular models. (**A**) Experimental approach to establish PCa resistance in a mouse model and to derive castration-resistant but ENZ-sensitive, and ENZ-resistant cell lines. Cx: surgical castration, Tx: ENZ treatment. (**B**) Viable cell count assay on VCaP^CRPC^ and VCaP^ER^ performed with and without increasing quantities of ENZ. *n* = 4 biological replicates. (**C**) Western blots showing expression of androgen receptor (AR) and resistance-specific splice variant AR-V7 in VCaP^CRPC^ and VCaP^ER^ (left). Quantifications of Western Blot signal intensity relative to VCaP^CRPC^ and normalized to total protein (right). *n* = 3 biological replicates. (**D**) Schematic for analysis of transcriptome, translatome and proteome in PCa cell lines: Transcriptome and proteome from VCaP, VCaP^CRPC^ and VCaP^ER^ and translatome from VCaP^CRPC^ and VCaP^ER^. (**E**) Heatmap and (**F**) boxplots of correlation coefficients for VCaP^CRPC^ and VCaP^ER^ proteomes with proteomes from patient samples. BPH: Benign prostate hyperplasia. T1C: tumor not detectable by palpation but detected upon needle biopsy. T2: tumor detectable by palpation but confined to the prostate. CRPC: Castration resistant prostate cancer. *P-value < 0.05; **P-value < 0.01; ***P-value < 0.001; ns indicates a non-significant *P*-value.

All animal procedures were performed according to Canadian Council on Animal Care guidelines and with approval of the Animal Care Committee of the University of British Columbia (protocol # A12-0210). One million VCaP cells were inoculated on both flanks of 6-week-old male athymic nude mice (Harlan Sprague-Dawley, Inc). Two weeks later, when tumors reached an approximate volume of 200 mm^3^, mice were surgically castrated. Castration resistance subsequently developed and when these tumors were growing beyond their pre-castration size, tumors were freshly harvested, washed, passaged and isolated from stromal cells in DMEM with 10% fetal bovine serum. Among several tumors concurrently passaged this way, cells termed VCaP^CRPC^ were selected for further experiments. Mice with castration-resistant tumor were then force-fed with 10 mg/kg ENZ (or vehicle) 5 days per week until tumor recurrence, at which point cells termed VCaP^ER^ were isolated as previously described and maintained in medium supplemented with 10 μM ENZ. This is summarized in Figure 1A.

### Cell culture and drugs

VCaP and LNCaP cell lines were obtained from ATCC. All cell lines were cultivated at 37°C with 5% CO_2_. LNCaP and MR49F (ENZ resistant cells derived from LNCaP) were cultivated in RPMI 10% FBS whereas VCaP, VCaP^CRPC^ (castration-resistant cells derived from VCaP) and VCaP^ER^ (ENZ-resistant cells derived concomitantly from VCaP^CRPC^) were cultivated in DMEM 15% FBS with 1 mM sodium pyruvate. ENZ-resistant cell lines (i.e. MR49F and VCaP^ER^) were maintained in 10 μM ENZ. ENZ was purchased from MedChemExpress (Cat. No.: HY-70002).

### Proliferation of ENZ resistance cell model

Cell count analyses were performed to assess the effect of ENZ treatment on cell proliferation and growth. VCaP^CRPC^, VCaP^ER^, LNCaP and MR49F cells were seeded at a density of 25 000 cells per well in 24-well plates, as technical triplicates, and allowed to attach and grow for 2 days. VCaP^CRPC^ and VCaP^ER^ were seeded in Geltrex™-coated plates, while LNCaP and MR49F were seeded without coating. Cells were treated without ENZ or with ENZ at concentrations of 5 or 10 μM. After, 3, 6, 9 and 12 days of treatment, images were taken and cells were detached using TrypLE (ThermoFisher Scientific; 12604021) and resuspended in PBS. Cells were stained with Trypan blue and viability was assessed by counts using a Countess automated cell counter (Thermo Fisher Scientific). Counts were performed twice for each technical replicate. Experiment was repeated as four biological replicates. Significant differences in viable cell counts between conditions were assessed by analysis of variance (ANOVA) followed by Tukey's honest significant difference test.

### Overexpression of gene candidates in VCaP^CRPC^ and LNCaP cells

VCaP^CRPC^ and LNCaP cells were lipotransfected with polycistronic vectors carrying doxycycline (DOX)-inducible TetO promoters (min.CMV) driving expression of gene candidates (Tg: transgene). Coding sequences for mRNA transcripts (Ensembl transcripts for *OCIAD1*: ENST00000264312.12, *GDAP1*: ENST00000220822.12, *PQBP1*: ENST00000447146.7, *RAB9A*: ENST00000464506.2 and *RAB3B*: ENST00000371655.4) and putative peptide-coding sequence for lncRNAs (Ensembl gene ID for *JPX*: ENSG00000225470, *CRNDE*: ENSG00000245694 and *LINC00467*: ENSG00000153363; Peptide sequences are available in Figure 4D) in the hg19 assembly; were cloned and overexpressed as transgenes. Cells were co-transfected with a second plasmid constitutively expressing a piggyBac transposase, which allows for random stable integration of transgenes into VCaP^CRPC^ or LNCaP genomes through piggyBac inverted terminal repeat (ITR) transposition. Vectors constitutively express a reverse tetracycline trans-activator (rtTA), which activates the TetO promoter upon addition of DOX, and a puromycin resistance gene (P) for selection. Transfected cells were selected for using 2 μg/ml and 750 ng/ml puromycin for VCaP^CRPC^ and LNCaP, respectively. Selection was maintained for 7 days. An IRES-controlled mCherry reporter gene downstream of the transgene enables monitoring the transgene RNA expression after DOX induction.

Cell lines were seeded at 25 000 cells/24-well dishes. After two days, induction of transgenes was performed by addition of 250 ng/ml DOX (Sigma-Aldrich #D9891-1G). Cells were treated with or without ENZ at 5 μM. Medium was changed, and pictures taken every three days. Viable cells were counted at day 12 for VCaP^CRPC^/VCaP^ER^ and day 9 for LNCaP/MR49F. Counts were performed as before; refer to the ‘Proliferation of ENZ resistance cell model’ section. For each sample, cell counts were normalized as log2(Fold change) of the average count of the corresponding untreated control condition. Points on the box plots represent three biological replicates and 2–3 technical replicates. Medians of control and enzalutamide-treated conditions were compared together to obtain a Δmedian and significant differences between two conditions were assessed via two-tailed T-tests assuming unequal variances.

### Preparation of cell pellets for downstream analyses

VCaP^CRPC^, VCaP^ER^, LNCaP and MR49F cells were grown as described above, in 100-mm tissue culture dishes to ∼80% confluence, for downstream analyses (Please refer to sections below). VCaP^ER^ and MR49F were grown with 10 μM ENZ.

### Mass spectrometry (MS) analysis

#### Sample preparation

Protein pellets from three biological replicates of VCaP, VCaP^CRPC^ and VCaP^ER^, and from one replicate of MR49F were resuspended in 100 μl of 50 mM ammonium bicarbonate, 0.5% deoxycholate and sonicated on ice with a microprobe Sonic Dismembrator 550 (Fisher Scientific) as follow: 20 × 1 s at power 2 followed by 5 × 3 s at power 4. The extract was centrifuged at 20 817 × *g* for 15 min at 4°C. The supernatants were transferred to new tubes and precipitated with acetone. The protein pellets were then resuspended in 100 μl of 500 mM triethylammonium bicarbonate, 0.5% deoxycholate. Protein concentrations of each sample was determined by colorimetric Bradford assay.

#### Tryptic digestion and TMT labeling

10 μg of each sample was used for TMT labeling (Thermo Fisher Scientific). Proteins were denatured for 5 min at 95°C and then reduced with 50 mM TCEP for 30 min at 37°C before being alkylated with 100mM iodoacetamide for 30 min at room temperature in the dark. Samples were digested with 0.5 μg of trypsin (V5111; Promega) for ∼15 h at 37°C. After digestion, peptides were acidified to precipitate the deoxycholate and then purified with homemade C_18_ Stage-Tip before being lyophilized. The now dried peptides were dissolved in 30 μl of 100 mM triethylammonium bicarbonate and labeled with TMT 10-plex reagent (Thermo Fischer Scientific). Labeling was performed for 1 hour at room temperature and the reaction quenched with hydroxylamine for 15 min. The now labeled peptides were combined in one tube and speedvac to dryness without heat. Samples were cleaned up using solid-phase HLB cartridge (Water Corp.) before being speedvac to dryness.

#### High pH: reverse phase fractionation

Peptides were fractionated into 14 fractions using a high pH (pH 10) reversed-phase chromatography method using an Agilent 1200 HPLC system as previously described (32). The final fractions were dried and resuspended in 0.1% formic acid before mass spectrometry analysis.

#### Mass spectrometry

Approximately 1 μg of each fraction was injected and separated by online reversed-phase (RP) nanoscale capillary liquid chromatography (nanoLC) and analyzed by electrospray mass spectrometry (ESI MS/MS). The experiments were performed with a Dionex UltiMate 3000 nanoRSLC chromatography system (Thermo Fisher Scientific/Dionex Softron GmbH, Germering, Germany) coupled to an Orbitrap Fusion mass spectrometer (Thermo Fisher Scientific, San Jose, CA, USA) equipped with a nanoelectrospray ion source. Peptides were trapped at 20 μl/min in loading solvent (2% acetonitrile, 0.05% TFA) on a 5 mm × 300 μm C_18_ PepMap cartridge pre-column (Thermo Fisher Scientific/Dionex Softron GmbH, Germering, Germany) for 5 min. Then, the pre-column was switched online with a 75 μm × 50 cm Acclaim PepMap100 C_18_—3 μm column (Thermo Fischer Scientific/Dionex Softron GmbH, Germering, Germany) and the peptides were eluted with a linear gradient from 5–40% solvent B (A: 0.1% formic acid, B: 80% acetonitrile, 0.1% formic acid) in 90 min at 300 nl/min. Mass spectra were acquired using a data dependent acquisition mode using Thermo XCalibur software version 3.0.63. Synchronous Precursor Selection-MS3 acquisition mode was used for this analysis. Full scan mass spectra (380–1500 *m*/*z*) were acquired in the Orbitrap at a 60 000 resolution and using an AGC target of 2e5, a maximum injection time of 50 ms. Internal calibration, using lock mass on the *m*/*z* 445.12003 siloxane ion, was used. Precursors for MS2/MS3 analysis were selected using a TopSpeed of 3 s. The most intense precursor ions were isolated in the quadrupole at 0.7 *m*/*z*, fragmented with 35% CID and the fragments detected in the ion trap. Following acquisition of each MS2 spectrum, an MS3 acquisition was performed by isolating of multiple MS2 fragment ions with a multi-notch isolation waveform (33). MS3 analysis was detected in the Orbitrap at a 60 000 resolution after 45% HCD, with an AGC target of 1e5 and a maximum injection time of 120 ms. Dynamic exclusion of previously fragmented peptides was set for a period of 20 s and a tolerance of 10 ppm.

#### Data analysis for mass spectrometry-based proteome quantification

Spectra acquired were processed using ProteomeDiscoverer 2.2 (Thermo). Files were searched against Uniprot (34) *Homo sapiens* protein database (93634 entries). Trypsin was set as enzyme and two missed cleavages were allowed. Deamidation (N, Q), oxidation (M), were set as dynamic modifications and carbamidomethylation (C), and TMT10-plex label (N-ter, K) were set as static modifications. Mass search tolerance were 10 ppm and 0.6 Da for MS and MS/MS respectively. For protein validation, a maximum False Discovery Rate of 1% at peptide and protein level was used based on a target/decoy search. MS3 spectra were used for quantification, with an integration tolerance of 10 ppm. Unique and razor peptides are considered for protein quantification and isotopic correction is applied on reporters. Data normalization was performed on total peptide amount. Peptides and protein result tabs were exported in Excel and means of three replicates per group were calculated. A fold change was calculated between the means of VCaP^ER^ and VCaP, VCaP^CRPC^ and VCaP, or VCaP^ER^ and VCaP^CRPC^. Proteins or peptides with variations >1.25 fold with a *P*-value ≤0.05 (or 0.1 for less stringent analysis in Supplementary Figure S4 C-D) in either their VCaP^ER^/VCaP or VCaP^ER^/VCaP^CRPC^ fold changes were considered as significant (either up or downregulated), so long as no opposite variation was found in these two.

#### Comparison of proteome quantifications to ENZ-resistance models and patient proteomics datasets

Average protein abundance counts for replicates for VCaP^CRPC^ and VCaP^ER^ MS were compared to the MR49F proteome through Spearman correlations. Expression of proteins with increased abundance in VCaP^ER^ or in VCaP^CRPC^ was assessed in MR49F, and a two-tailed Student's *t*-test was used to highlight significant differences from the whole distribution of MR49F proteins, to determine if VCaP^ER^’s or VCaP^CRPC^’s upregulated proteins are also upregulated in other models. Each replicate of VCaP^CRPC^ and VCaP^ER^ MS was also individually compared to patient proteomics data from Latonen *et al.* (35) using Pearson correlations. Correlation coefficients were compared to VCaP^CRPC^ and VCaP^ER^ for each group of sample grades (BPH, T1C, T2 and CRPC) using two-tailed Student's *t*-tests.

#### Construction of biological networks

Interactions among identified differentially expressed proteins were mapped with the STRING database (36). Two protein-protein interaction (PPI) networks were constructed (for upregulated and downregulated proteins) for experimentally validated interactions and databases with a required interaction score a 0.9. Subsequently, the PPI networks were imported into Cytoscape (37) using stringApp (38). The identified hub genes related GO terms were used to construct a complete PPI network.

#### Gene set enrichment, modules and network analysis

Gene Set Enrichment Analysis (GSEA) was performed with Broad Institute's GSEA software (v4.1.0) (39,40). Expression data sets were created as text files according to GSEA specifications. We computed overlaps with the C2.cp.kegg (curated gene sets) and C5.go, C5.go.bp, C5.go.cc and C5.go.mf (GO gene sets) collections. Gene set permutations were performed 1000 times per analysis. An FDR <0.1 was set as cut-off for significant enrichment.

#### Western blot for AR, AR-V7, 4E-BP1 and eIF2α

VCaP^CRPC^ and VCaP^ER^ cells were lysed in RIPA buffer containing protease and phosphatase inhibitors. Proteins were separated on SDS-PAGE and transferred to nitrocellulose membranes (Bio-Rad). Membranes were blocked in PBS containing 0.1% Tween-20 and 5% non-fat milk or 5% BSA for 30 min, followed by incubation with primary antibodies overnight at 4°C. The following antibodies were used: anti-AR and anti-AR-V7 (Cell Signaling Technology (Beverly, MA, USA)) diluted 1:1000. Anti-eIF2α diluted 1:1000, (#9722, Cell Signaling Technology), anti-phospho-eIF2α diluted 1:500 (#9721, Cell Signaling Technology), anti-4EBP1 diluted 1:5000 (#9644, Cell Signaling Technology) and anti-phospho-4EBP1 diluted 1:1000 (#9456, Cell Signaling Technology). Membranes were then incubated with HRP-conjugated secondary antibodies (Jackson ImmunoResearch Laboratories Inc, West Grove, PA) 1 h in PBST 5% milk (1:10 000). Immunoblots were visualized using enhanced chemiluminescence (ECLPlus, Perkin-Elmer). TCE-UV stain-free total protein visualization was used as loading control (41).

#### Ribopuromycylation assay

Anti-puromycin antibody was obtained from EMD Millipore (Merck, Germany). Puromycin was purchased from Sigma (St Louis, MO) and dissolved in water as a 25 mg/ml stock solution, aliquoted and stored at −20°C. Cells were plated to reach a confluency of ∼80% the day of the treatment. For puromycylation assay, cells were labelled with 10 μg/ml of puromycin for 5 min. Cells were collected and proteins were separated on SDS-PAGE and transferred to nitrocellulose membranes (Bio-Rad). Membranes were blocked in PBS containing 0.1% Tween-20 and 5% non-fat milk or 5% BSA for 30 min, followed by incubation with primary antibodies overnight at 4°C. Anti-puromycin was diluted 1:25 000 (EMD Millipore). After incubation with horseradish peroxidase coupled secondary antibodies at room temperature for 1 h, immunoblots were visualized using enhanced chemiluminescence (ECLPlus, Perkin-Elmer). Coomassie staining of total proteins was used as loading control.

#### Mitochondrial metabolism analysis

An extracellular Flux Analyzer XFe96 (Agilent/Seahorse Bioscience) was used to analyze live-cell mitochondrial respiration. VCaP^CRPC^ and VCaP^ER^ were seeded in a poly-l-lysine coated 96-well XFe96 plate (15 000 cells/well). After 48h treatment, oxygen consumption rate (OCR) was measured using standard protocols for PCa cells (42–44). At the end of the assay, cells were harvested and counted using CyQUANT to normalize for cell number. Average and SEM of one representative experiments, with three replicates/condition, out of three independent experiments is shown. Student's *t* test was performed to evaluate statistical significance.

#### DNA extraction and qPCR for mt/N DNA ratios

VCaP^CRPC^ and VCaP^ER^ were seeded in a 6-well plate at 500 000 cells/well. After 72 h of growth, DNA was purified using the DNeasy Blood and Tissue kit (QIAGEN). Mitochondrial/nuclear (Mt/N) DNA ratios were determined by qPCR, using the LUNA Universal qPCR reagents (New England Biolabs). *CYTB* and *ND1* genes were used to quantify mitochondrial DNA, and *EP300* and *HPCAL4* for nuclear DNA, as previously described (45) (see Supplemental Table 6). Results are shown as the average and SEM of three independent experiments, each consisting of three biological replicates, and statistical significance was evaluated using a Student's *t* test.

#### Polysomal profiles, isolation of polysome-associated RNAs and analysis

VCaP^CRPC^, VCaP^ER^, LNCaP and MR49F cells were scraped in 1 mL of polysomal buffer (20 mM Tris, pH 7.5, 150 mM NaCl, 1.25 mM MgCl_2_, 5 U/ml RNasin, cOmplete™ EDTA-free Protease Inhibitor Cocktail (Roche, Indianapolis, IN) and 1 mM dithiothreitol), and Nonidet *P*-40 was added to a final concentration of 1% for lysis, 15 min on ice. Extracts were clarified by centrifugation at 12 000 × g for 20 min at 4°C. RNA concentration was measured by spectrophotometry and ∼20 OD_260_ units of RNA were loaded onto a 15–55% sucrose gradient. The gradients were centrifuged for 2.5 h at 37 000 rpm (223 000 × g) (SW 40 TI Beckman rotor) and then placed on an Automated Density Fractionation System (Teledyne Isco, Density Gradient Fractionation System) to collect fractions. Each fraction was collected into individual tubes with continuous monitoring of absorbance at 254 nm. Absorbance was recorded on chart paper to generate polysomal charts. RNA from each fraction was extracted by phenol–chloroform extraction and fractions corresponding to light or heavy polysomes were respectively pooled together.

For quantification of area-under-the-curves for polysome graphs, polysome profiles from four biological replicates for VCaP^CRPC^ and VCaP^ER^, or three biological replicates for LNCaP and MR49F were scanned and digitized. For each biological replicate, polysome profiles for VCaP^CRPC^ and VCaP^ER^ were overlayed to assess differences. Area-under-curve was calculated using ImageJ, as number of pixels for sub-polysomal fractions (i.e. area corresponding to peaks of the 40S, 60S and 80S ribosomal sub-units) and for polysomal fractions (i.e. area from the first polysomal peak to the twelfth polysomal peak, as reported by Haneke *et al.* (46)). Lower limit of the Y axis values was defined by the lowest point of the graph. Polysomal fractions area was normalized to the sub-polysomal area and presented as fold change over area corresponding to VCaP^CRPC^. Significant difference between VCaP^CRPC^ and VCaP^ER^ was determined via non-parametric *t*-test.

### Transcriptome and translatome analysis

#### RNA extraction

For analysis of the transcriptome, RNA was extracted from VCaP, VCaP^CRPC^, VCaP^ER^, LNCaP and MR49F with TRIzol reagent (Life Technology; 15596018). For polysome profiling, heavy polysomal RNA was prepared from VCaP^CRPC^, VCaP^ER^, LNCaP and MR49F polysomal fractions by phenol-chloroform extraction.

#### RT-qPCR

RNA was purified from five biological replicates of VCaP^CRPC^ and VCaP^ER^ or from 2 biological replicates of LNCaP and MR49F, and DNase treated and purified via RNA clean-up columns (Zymo Research, RNA Clean & Concentrator-5 (with DNase), VWR; 76020-604). RNA was then reverse-transcribed for RT-qPCR using iScript™ gDNA Clear cDNA Synthesis Kit (Bio-Rad #1725035) following the manufacturer's instructions. All RT-qPCR analyses were performed on CFX384 Touch Real-Time PCR Detection system instrument (Bio-Rad) using SYBR Select Master Mix (ThermoFisher Scientific; 4472919). The reaction mix (10 μl) was prepared according to the manufacturer's instructions, using each primer (see Supplemental Table S3) in a final concentration of 300 nM. The cycling conditions were set according to the manufacturer's instructions, using a primer annealing temperature of 58°C. All RT-qPCR reactions were performed in technical triplicates. Translation efficiency was calculated by normalizing the relative fold-change of heavy polysomal RNA extracted from polysome profiling, to the RNA expression in total RNA. This was performed separately in VCaP^CRPC^, VCaP^ER^, LNCaP and MR49F, and normalized to expression of the *RPS13* housekeeping gene. Average of biological and technical replicates is shown with error bars representing SEM. Significant differences in translation efficiency between VCaP^CRPC^ and VCaP^ER^, or between LNCaP and MR49F was ascertained by two-tailed *t*-tests assuming unequal variances. Refer to Supplemental Table S3 for primer sequences.

#### RNA sequencing library preparation and analysis

For samples used in RNA sequencing (two biological replicates of total RNA from VCaP, VCaP^CRPC^ and VCaP^ER^, and from heavy polysomal RNA from VCaP^CRPC^ and VCaP^ER^). RNA quality was verified with the TapeStation 4200 (Agilent Technologies). RNA libraries were made from 0.2 ug of RNA in accordance with the TruSeq stranded mRNA kit protocol (Illumina; # 20020594) and TruSeq RNA Single Indexes Set A and B (Illumina, # 20020492 and 20020493). Library qualities and sizes were checked with the TapeStation 4200 and then quantified using the KAPA Library Quantification Kit for Illumina platforms (Kapa Biosystems). Libraries were sequenced on the Illumina NextSeq500 sequencer to a depth of about 50 millions of 75-bp pair reads per library.

The reads were aligned to the GRCh37 human genome (Ensembl release 75) by STAR (v2.7.5) (47). Read alignments were merged and disambiguated, and a single BAM (Binary Alignment Mapped) file output per library or sample was used. BAM files were then additionally filtered to remove reads with a mapping quality (MAPQ) <13, and all ribosomal and mitochondrial RNA reads. Alignments were assembled using Cufflinks (v2.2.1) (48) using the –g parameter to construct a genome annotation file against the reference gene model (Ensembl release 75) and to identify novel transcripts. Raw read counts were obtained by mapping reads at the gene level using the Cufflinks assembled transcript annotation file with the featureCounts tool from the SubRead package (version 2.0.0) (49) using the exon counting mode. EdgeR R-package (v3.12.1) (50) was then used to normalize the data, calculate transcript abundance (as counts per million reads (CPM)) and perform statistical analysis. Briefly, a common biological coefficient of variation (BCV) and dispersion (variance) was estimated based on a negative binomial distribution model. This estimated dispersion value was incorporated into the final EdgeR analysis for differential gene expression, and the generalized linear model (GLM) likelihood ratio test was used for statistics, as described in EdgeR user guide. Genes were considered as significantly up or downregulated in either total or polysome-bound RNAseq if their fold change between VCaP^ER^ and VCaP^CRPC^ were superior to 1.25 fold, with a FDR ≤0.05. Concordance of the transcriptome and the translatome was assessed via Pearson correlations. All statistical analyses and data visualization were done in R using R basic functions and the following packages: gplots (3.1.1), stats4 (3.5.1), plyr (1.8.4), dplyr (0.8.1) and ggplot2 (3.1.1).

#### Translation efficiency (TE) analysis

We assessed translation efficiency for each transcriptome replicate by comparing separately to each translatome replicate, and vice-versa. Hence, we calculated TE for each gene and each comparison of replicates in VCaP^ER^ and VCaP^CRPC^ as follows: ${\rm TE} = \frac{{{\rm Abundance}\,{\rm in}\,{\rm translatome}\,( {{\rm CPM}} )}}{{{\rm Abundance}\,{\rm in}\,{\rm transcriptome}\,( {{\rm CPM}} )}}$. An average was calculated for each comparison in VCaP^ER^ and VCaP^CRPC^ and variation between these two lines was calculates as a TE ratio. Significant differences were assessed with via Student's T-test adjusted for multiple comparisons with a 10% FDR. TE ratios >1.25 with adjusted *P*-values ≤0.05 were considered significant.

In parallel, TE ratios were also calculated with two other complementary methods. First, we employed the DeltaTE software (51) using the base parameters. Here, we set the ‘batch’ parameter to allow for each transcriptome replicate to be separately analyzed against each translatome replicate, and vice-versa.

Next, we assessed TE ratios using anota2seq (52). We used the anota2seqDataSetFromMatrix function to initialize an Anota2seqDataSet object from out data, with the following arguments: dataType = ‘RNAseq’, filterZeroGenes = TRUE, normalize = TRUE, transformation = ‘TMM-log2’, varCutOff = NULL. The phenoVec argument was set as to allow, as before, for each transcriptome replicate to be separately analyzed against each translatome replicate, and vice-versa. Next, we assessed the model assumptions with anota2seqPerformQC using the following arguments: generateSingleGenePlots = TRUE, useRVM = TRUE, and normality of the residuals from the linear regression were checked with the anota2seqResidOutlierTest function. Finally, the anota2seqAnalyze function was run with the following parameters: analysis = c(‘translation’, ‘buffering’, ‘total mRNA’, ‘translated mRNA’) and genes were categorized as either mode of gene expression change (changes in mRNA abundance, changes in translational efficiency, or buffering where constant levels of translating RNAs are maintained despite total RNA levels being altered). Genes with an adjusted *P*-value ≤0.05 were considered significant.

Genes with significant TE ratios were separated according to their gene type. Expected and observed frequencies were compared via Chi-squared tests, to assess enrichment/depletion of certain gene types in polysomal RNA. Expected values under null hypothesis were calculated as:

$$\begin{equation*}\frac{{{\rm{Sum}}\,{\rm{of}}\,{\rm{all}}\,{\rm{genes}}\,{\rm{of}}\,{\rm{the}}\,{\rm{same}}\,{\rm{type}}\,{\rm{across}}\,{\rm{samples}}\,{\rm{X}}\,{\rm{Sum}}\,{\rm{of}}\,{\rm{all}}\,{\rm{genes}}\,{\rm{in}}\,{\rm{the}}\,{\rm{same}}\,{\rm{sample}}}}{{{\rm{Total}}\,{\rm{sum}}\,{\rm{of}}\,{\rm{all}}\,{\rm{genes}}\,{\rm{across}}\,{\rm{all}}\,{\rm{samples}}}}\end{equation*}$$

Differences with *P* < 0.05 were considered statistically significant.

#### Analysis of alternative splicing events

BAM files from the transcriptome and the translatome RNA-sequencing experiments were compared for differential splicing events using rMATS (53,54), using only reads that span splicing junctions (JC analysis) with the following arguments: –variable-read-length –novelSS –mel 1000. Events with a *P*-value <0.05 and an inclusion level difference >0.1 between the transcriptome and translatome samples for either VCaP^CRPC^ or VCaP^ER^ were considered significant. Events were classified as to whether they occurred in VCaP^CRPC^ exclusively, in VCaP^ER^ exclusively, in both VCaP^CRPC^ and VCaP^ER^ and varying in the same direction (e.g. an exon more often skipped in polysomal RNA in both cell lines), or in both VCaP^CRPC^ and VCaP^ER^ varying in opposite directions (e.g. an exon more often included in VCaP^CRPC^ polysomes, but skipped in VCaP^ER^ polysomes). Events corresponding to exon skipping or mutually exclusive exons were combined as Alternative exon choice (AEC) events. Alternative 3’ and 5’ splice site choice events were combined as Alternative splice site (ASS). Significant AEC, ASS and intron retention (IR) events were quantified and separated for whether they occurred in mRNAs or lncRNAs. Significant enrichment for an event type in the translatome versus transcriptome for VCaP^CRPC^ or VCaP^ER^ was determined by chi-squared tests comparing expected and observed frequencies for events. Expected values under null hypothesis were calculated as:

$$\begin{equation*}\frac{{\left( {{\rm{Sum}}\,{\rm{of}}\,{\rm{all}}\,{\rm{genes}}\,{\rm{of}}\,{\rm{the}}\,{\rm{same}}\,{\rm{type}}\,{\rm{across}}\,{\rm{samples}}} \right){\rm{X}}\left( {{\rm{Sum}}\,{\rm{of}}\,{\rm{all}}\,{\rm{genes}}\,{\rm{in}}\,{\rm{the}}\,{\rm{same}}\,{\rm{sample}}} \right)}}{{{\rm{Total}}\,{\rm{sum}}\,{\rm{of}}\,{\rm{all}}\,{\rm{genes}}\,{\rm{across}}\,{\rm{all}}\,{\rm{samples}}}}\end{equation*}$$

Differences with *P*-value < 0.05 were considered statistically significant. Events occurring in the lncRNA *JPX* were compiled with featureCounts (49) using the following arguments: -T 8 -O -J –fraction –largestOverlap -t exon -g transcript_name_id. *JPX* transcript annotations were obtained from Ensembl's GRCh38 release 107 and are provided as Supplementary material 1. featureCounts was restricted to the *JPX* locus. Reads corresponding to transcripts with altered translation initiation site and Kozak sequence for *JPX*’s putative peptide were grouped together and counted (refer to Supplementary Table S11). Significant differences in abundance for transcripts with selected splicing events between VCaP^CRPC^ and VCaP^ER^ were assessed via two-tailed T-tests assuming unequal variances. Translation initiation site (TIS) scores were predicted with TISpredictor (55).

#### cBioPortal analysis

Integrative analysis of publicly available PCa patient data was performed using the public databases of cBioPortal (https://www.cbioportal.org/) (56). We focused our analysis on data generated by the TCGA research network (The Cancer Genome Atlas, https://www.cancer.gov/tcga). We selected all studies implicated in PCa (25 in total as of 2022). We selected samples where mRNA expression was available (266 samples). For each candidate gene, samples were separated into mRNA expression quartiles (in FPKM) and association towards clinical attributes (AR score—PolyA, polyA ARV7 SRPM spliced reads per million, NEPC score—PolyA, ETS Fusion—SEQ) was assessed.

## RESULTS

### Development and characterization of an ENZ-resistant PCa progression model

With the advent of potent AR-antagonists such as ENZ as first line therapy for castration-resistant prostate cancer (CRPC) patients, a few ENZ-resistant cellular models have been described (57,58). Among the first and most widely characterized ENZ-resistant cells were MR49F (31,59,60), generated through serial passage of LNCaP cells (androgen-sensitive prostate adenocarcinoma cells) in ENZ-treated mice. To develop a complementary model that contains a concomitantly passaged castration-resistant control from human PCa cells (which is not available for the LNCaP and MR49F cell lines), we used a similar approach with the wild-type AR VCaP cell line (59) (i.e. AR is mutated in LNCaP cells (61)) (Table 1). VCaP cells were inoculated in male athymic nude mice (Figure 1A); mice were surgically castrated, and cells termed VCaP^CRPC^ were derived from tumors resistant to castration. In parallel, mice with castrate-resistant tumors were treated with ENZ until tumor regrowth, at which time the VCaP^ER^ cell line was established.

**Table 1.** Characteristics of AR in PCa cell lines

Cell line	Androgen receptor	AR-V7 splice variant levels
VCaP	Wild-type	Low-moderate
VCaP^CRPC^	Wild-type	Low-moderate
VCaP^ER^	Wild-type	High
LNCaP	T878A mutant	Very low
MR49F	T878A and F876L mutant	Very low

Summary of androgen receptor status of ENZ-resistance models.

We first confirmed acquisition of ENZ-resistance in VCaP^ER^ cells using cell survival assays, in the presence or absence of ENZ. After 12 days of ENZ treatment, a significant reduction in the number of viable cells, changes in morphology (i.e. going from dome-like to flattened colonies), and increased cell death was observed for VCaP^CRPC^ (Figure 1B; Supplemental Figure S1A and B). This was recapitulated in the ENZ-sensitive LNCaP cells (Supplemental Figure S1C and D). Comparatively, VCaP^ER^ and MR49F were unaffected in the usual clinical concentration for ENZ (62) (Figure 1B and Supplemental Figure S1). We next verified whether our ENZ-resistant VCaP^ER^ cell line recapitulated key characteristics of clinical ENZ-resistant prostate cancer. We assessed expression of the androgen receptor, a frequently overexpressed driver of prostate cancer, and of its AR-V7 splice variant, which is associated with clinical ENZ-resistance (58,63,64). This variant encodes a truncated protein that lacks the C-terminal ligand-binding domain but retains the N-terminal domain and therefore constitutively activates downstream target genes, which are involved in PCa progression (65,66). AR-V7 expression is generally higher in advanced PCa and has previously been linked to ENZ resistance (58). Correspondingly, we found that VCaP^ER^ displayed increased AR and AR-V7 splice variant expression (Figure 1C; Table 1, Supplemental Figure S2. A-B), indicating that our ENZ-resistance model recapitulates key characteristics of clinical ENZ-resistant prostate cancer.

To understand how transcription and translation are coordinated during acquisition of ENZ-resistance in PCa, we analyzed the transcriptome (i.e. Poly(A)+ RNA-seq), translatome (i.e. Heavy polysomal RNA-seq) and proteome (i.e. total proteins quantified by mass spectrometry (MS)) from the VCaP^CRPC^ and VCaP^ER^ cell lines, and included the transcriptome and proteome from the parental VCaP cell line as an additional control (Figure 1D). We validated that the proteins highly expressed in VCaP^ER^ were linked to ENZ-resistance globally and not only specific to our model by comparing to the proteome of previously established ENZ-resistant MR49F cell line, which was independently derived from the parental LNCaP cells (31). We show that proteins highly expressed in VCaP^ER^ correspond well with those highly expressed in MR49F, which was not the case for downregulated proteins (i.e. highly expressed in VCaP^CRPC^) (Supplemental Figure S3A and B).

We next sought to determine if the generated proteomics data truly recapitulated the determinants of clinical PCa, to further validate our resistance model. By comparing to patient proteomics data (35), we find that VCaP^CRPC^ and VCaP^ER^ proteomes correlate poorly with benign prostate hyperplasia (BPH) but show an increase in correlations with gradually increasing PCa grade (Figure 1E and F). Proteomics data from our VCaP^CRPC^ PCa models, and to a lower extent VCaP^ER^, correlate well with castration resistant cancer (CRPC) patient samples. Interestingly, VCaP^ER^ data correlates less with low-grade untreated PCa patient samples (T1C and T2, localized PCa) when compared to VCaP^CRPC^ (Figure 1F, Supplemental Figure S3C), which may indicate that the VCaP^ER^ proteome is more akin to advanced, treatment resistant PCa. Hence, here we present a novel model of PCa ENZ-resistance with high concordance to the main clinical features of advanced PCa and high similarity to established patient sample proteomes.

### Increased mitochondrial activity and reduced translation in ENZ-resistant cells

**Figure 2.**
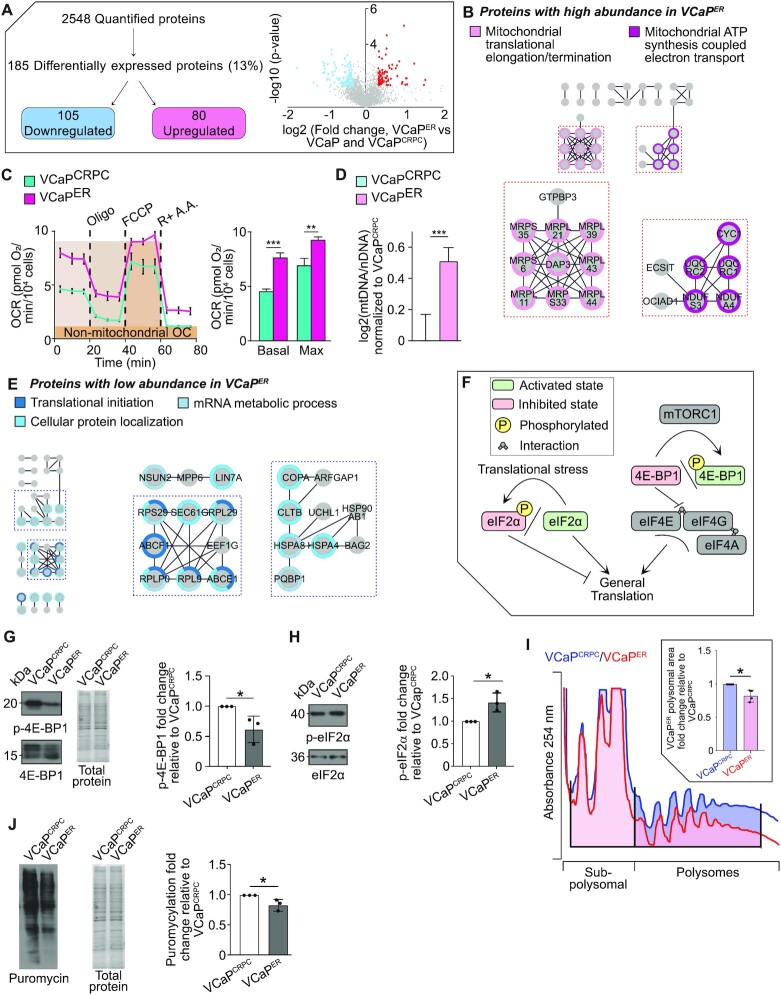
VCaP^ER^ are characterized by increased mitochondrial activity and decreased translation. (**A**) Mass spectrometry analysis showing differentially expressed proteins in VCaP^ER^ cells compared to VCaP^CRPC^ and VCaP (left). Volcano plot showing fold change between VCaP^ER^ cells and VCaP^CRPC^ (right). Significantly up- and downregulated proteins are marked by red and blue dots respectively. (**B**) Network analysis showing clusters formed by proteins highly expressed in VCaP^ER^ compared to VCaP^CRPC^. Main clusters are identified and highlighted in pink. (**C**) OCR of VCaP^ER^ and VCaP^CRPC^ cells during mitochondrial stress test (left) and basal and maximal OCR in VCaP^CRPC^ and VCaP^ER^ (right). Results are shown as the average and standard error of the mean (SEM) of 3 independent experiments performed in triplicates. (**D**) Mitochondrial DNA and nuclear DNA ratio in VCaP^CRPC^ and VCaP^ER^ cells. Results are shown as the logged average and SEM of 3 independent experiments performed in triplicates, normalized to VCaP^CRPC^. (**E**) Network analysis shows clusters formed by proteins lowly expressed in VCaP^ER^ compared to VCaP^CRPC^ and VCaP. Main clusters are identified and highlighted in blue. (**F**) Schematic of the effect of eIF2α and 4E-BP1 phosphorylation on translation. (**G**) Western-blot showing phosphorylation of 4E-BP1 and (**H**) eIF2α in VCaP^CRPC^ and VCaP^ER^ cell lines (left). Quantification of signal intensities (right), relative to VCaP^CRPC^. 4E-BP1 was normalized to total protein and p-eIF2α, to eIF2α. *n* = 3 biological replicates. (**I**) Polysomal profiles of VCaP^CRPC^ (blue) and VCaP^ER^ (red) (left) and quantification of area under curve of polysomal area (top right), normalized to sub-polysomal area and relative to VCaP^CRPC^. *n* = 4 biological replicates. (**J**) Puromycylation assay on VCaP^CRPC^ and VCaP^ER^ cell lines (left) and quantification (right) relative to VCaP^CRPC^ and normalized to total protein. *n* = 3 biological replicates. *P-value < 0.05

To investigate whether specific pathways or biological processes play a role in driving the emergence of ENZ-resistance in PCa, we inspected the differentially expressed proteins in VCaP^ER^ compared to VCaP and VCaP^CRPC^. Out of the 2548 identified proteins in our dataset, 185 were differentially expressed, with a significant increase in abundance for 80 proteins in VCaP^ER^, and a decrease for 105 (Figure 2A; Supplemental Figure S3D–F; Table S1). Moreover, expression of VCaP^ER^-enriched proteins was equivalent in both the parental VCaP and their castrate-resistant derivatives, VCaP^CRPC^ (Supplemental Figure S3E), suggesting that these genes do indeed correspond to a signature of enzalutamide resistant PCa cells.

Gene network analyses revealed that ENZ-resistance promoted up-regulation of two main protein clusters, corresponding to mitochondrial translation factors and electron transport (Figure 2B; Supplemental Figure S4A, C; Table S2). Previous work has demonstrated that AR activation stimulates mitochondrial respiration and biogenesis (42–44,67–69). Thus, AR blockade with ENZ was expected to decrease this mitochondrial response, while ENZ-resistance would be expected to restore increased mitochondrial activity in PCa cells. Therefore, to assess if these changes in mitochondrial protein levels were indicative of an impact of ENZ-resistance on cellular metabolism and mitogenesis, we conducted extracellular flux analysis to quantify cellular oxygen consumption rates (OCR) (Figure 2C). Our results showed that ENZ-resistant VCaP^ER^ demonstrate a significant increase of basal mitochondrial respiration compared to VCaP^CRPC^ (Figure 2C). Furthermore, following a mitochondrial stress test, VCaP^ER^ cells also exhibited increased maximal respiration capacity (Figure 2C; right panel). In addition, to assess the impact of ENZ-resistance on mitochondrial cell content, mitochondrial DNA over nuclear DNA (mtDNA/nDNA) ratios were measured (Figure 2D, Supplemental Tables S3 and S4). A significant increase in mtDNA/nDNA was observed in VCaP^ER^ compared to VCaP^CRPC^. Altogether, these findings indicate increased mitochondrial content and activity in VCaP^ER^, in agreement with proteomics analyses demonstrating increased abundance for mitochondrial translation machinery and proteins of the electron transport chain.

We also investigated proteins with decreased abundance in VCaP^ER^ compared to VCaP^CRPC^. Network analyses found protein clusters involved in regulation of cytoplasmic translation and mRNA metabolic processes (Figure 2E; Supplemental Figure S4B, D; Table S2). Various translation regulators such as eIF4B, ABCE1 and 4E-BP1, or ribosomal proteins, for example RPL29 or RPL9 which have been linked to malignant PCa and other cancers (7,70–75), were found in low abundance in VCaP^ER^ (Figure 2E, Supplemental Figure S4B, D). These results were corroborated by GSEA (Supplemental Figure S5; Table S5) and together, suggest that ENZ-resistance may affect translation. We further defined the network corresponding to mRNA metabolic processes through less stringent analyses, which revealed enrichment of several proteins linked to alternative splicing, such as PQBP1, SNRNP70, HSPA8 and LSM3/7 (76,77) (Supplemental Figure S4B, D). To understand how a decrease in the expression of translation factors in VCaP^ER^ could affect global translation levels, we assessed phosphorylation levels of 4E-BP1 and eIF2α, which represent two major factors implicated in regulating the initiation step of translation (78) (Figure 2F). We show that, in addition to the observed decrease in total levels of the 4E-BP1 protein, VCaP^ER^ also display significantly reduced normalized levels of phosphorylated 4E-BP1 (Figure 2G, Supplemental Figure S2A,C). Correspondingly, higher eIF2α phosphorylation is detected in VCaP^ER^ (Figure 2H, Supplemental Figure S2D) indicating a decreased translation initiation compared to VCaP^CRPC^.

To further confirm that VCaP^ER^ have reduced global translation as compared to VCaP^CRPC^, we quantified polysomes, which mostly reflect the proportion of translating ribosomes. We observed a significantly diminished area under the curve in the polysomal fractions isolated from VCaP^ER^, compared to those corresponding to VCaP^CRPC^ (Figure 2I; Supplemental Figure S6-B, Table S6). This indicates that the overall relative level of translating ribosomes is lower in VCaP^ER^, which is consistent with the previously observed downregulation of translation initiation (Figure 2G, H). We corroborated these results using (ribo)puromycylation assays, in which VCaP^ER^ displayed significantly reduced protein synthesis (Figure 2J, Supplemental Figure S2E). To determine whether downregulated translation is a general feature of ENZ resistance, rather than a cell line-specific effect, we investigated polysomal profiles of LNCaP and their ENZ-resistant related cell line, MR49F. We observe that MR49F, similar to VCaP^ER^, exhibit a lower area under the curve for polysomal fractions (Supplemental Figure S7, Table S6), suggesting that a decrease in translation may be characteristic of ENZ resistance in the broader term.

Together, these results suggest that ENZ-resistant PCa cells are defined by alterations in mitochondrial composition and activity, but also by a general downregulation of translation.

### Genes encoding highly translated RNAs in VCaP^ER^ are drivers of PCa ENZ resistance

**Figure 3.**
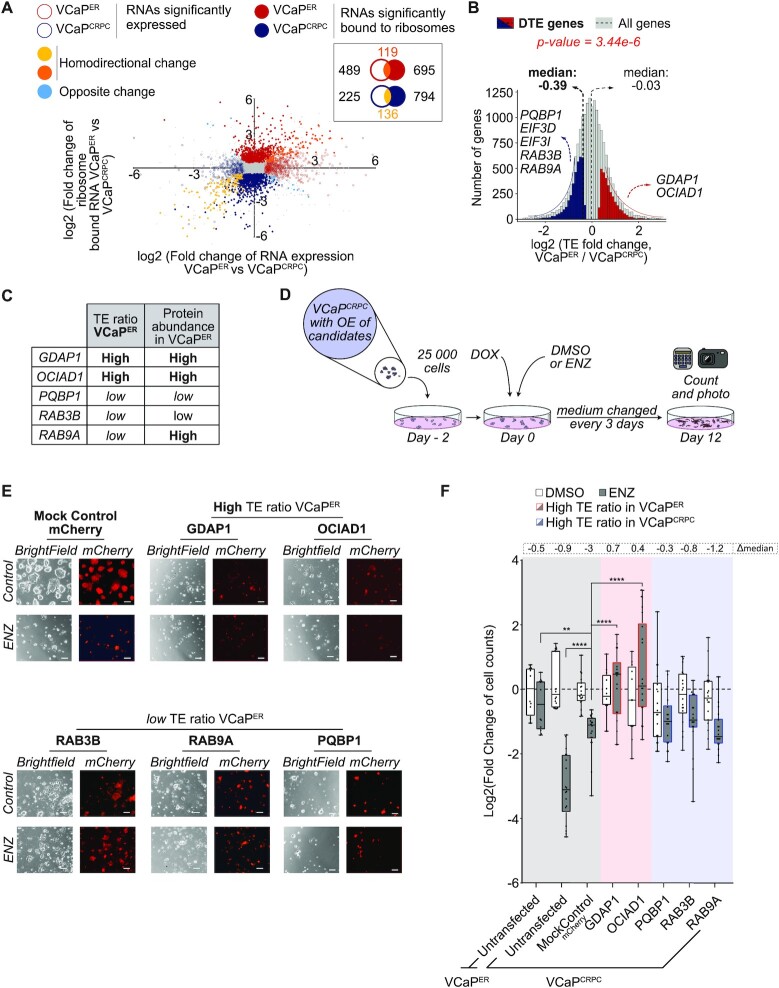
Highly translated genes in VCaP^ER^ are drivers of PCa ENZ resistance. (**A**) Scatterplot highlighting RNAs significantly increased or decreased in the transcriptome (empty circles) or translatome (full circles) in VCaP^ER^ (red) compared to VCaP^CRPC^ (blue). Significant genes are colored. *n* = 2 biological replicates for each condition. (**B**) TE ratios for all genes, and for genes with a significant differential TE ratio in VCaP^ER^ compared to VCaP^CRPC^ (red for higher and blue for lower TE ratio). Medians are indicated with dashed lines (thin line for all genes, bold line for genes with significant differences in TE ratio). (**C**) Table of gene candidates selected for additional studies. (**D**) Schematic of overexpression experiments for candidate genes. (**E**) Representative images of cell lines for GDAP1, OCIAD1, RAB3B, RAB9A and PQBP1. Scale bar: 100 uM. (**F**) Fold changes of viable cell counts for cell lines overexpressing candidate genes with ENZ treatment normalized to control without ENZ treatment. Differences between ENZ-treated and control in log2(fold changes) are indicated as Δmedian. *n* = 2 or 3 biological replicates. *P-value < 0.05; **P-value < 0.01; ***P-value < 0.001; ****P-value < 0.0001. Only significant comparisons are shown.

To better understand the mechanisms underlying the interplay of translation and development of drug resistance in PCa, we next investigated alterations in our transcriptome and translatome RNA-seq datasets. Analysis of the transcriptome revealed 608 genes with increased RNA abundance in VCaP^ER^ compared to VCaP^CRPC^, and 361 genes with low RNA abundance (Figure 3A; Supplemental Table S7). We also assessed RNA association to ribosomes by sequencing RNAs isolated from heavy polysomal fractions. This highlighted 814 RNAs with higher, and 930 with lower association to ribosomes in VCaP^ER^ compared to VCaP^CRPC^ (Figure 3A; Supplemental Table S7). While we do observe good correlation between the transcriptomes and translatomes of VCaP^ER^ and VCaP^CRPC^ in general (Supplemental Figure S8A-B), it was not always the case with top varying genes. In fact, 37% of significantly upregulated genes in VCaP^CRPC^ (136 out of 361) were also more associated to ribosomes, while only 20% (119 out of 608) of significantly upregulated genes in VCaP^ER^ also showed increased association to ribosomes (Figure 3A). Therefore, highly transcribed RNAs in VCaP^ER^ tend to have a lesser propensity to associate with ribosomes, which is consistent with our biochemical results from above (Figure 2G–I). This suggests potential perturbations of the translating RNA pool in ENZ-resistant cells, which are independent from RNA abundance. It also echoes the overall decrease in translation in ENZ-resistant cells, and potentially reflects a decrease in overall translational rate.

To assess the translation rate of RNAs, we normalized polysomal occupancy of an RNA to its total abundance, giving us a metric called translation efficiency (TE). We then calculated a TE ratio for individual genes by taking the fold change in TE values between VCaP^ER^ and VCaP^CRPC^ (Figure 3B; Supplemental Table S8). Consistent with the VCaP^ER^ translatome and proteome data, we observe a general decrease in TE ratios in VCaP^ER^ compared to VCaP^CRPC^ (i.e. less ribosome association of RNAs in VCaP^ER^ than in VCaP^CRPC^, with a median logged TE ratio of −0.39 for genes with a differential TE ratio) (Figure 3B). However, this decrease does not equally affect all RNAs, as we detect a small portion of RNAs for which association to ribosomes is instead increased in ENZ-resistant cells, and whose proteins were also upregulated in the VCaP^ER^ proteome (corresponding to 13% of proteins with increased abundance in VCaP^ER^, Supplemental Figure S9). This specific set of RNAs therefore escapes the translation downregulation observed in ENZ-resistant cells, resulting in increased protein abundance. We selected several candidate mRNAs with either increased or decreased TE in VCaP^ER^ for RT-qPCR validation. We selected mRNAs detected with a minimum of 5 CPM in transcriptome and translatome to ensure detection by RT-qPCR, and whose corresponding proteins were also differentially expressed. Changes in TE were confirmed for these candidates (Supplemental Figure S8C, Tables S3 and S4). We further validated our results assessing translation efficiency using available software, such as annota2seq and deltaTE (51,52). We observed high concordance between our method and the deltaTE software (Supplemental Figure S10), which further validates our analyses.

The next step was therefore to investigate whether the polysome-bound mRNAs encoding higher abundance proteins in VCaP^ER^ could act as molecular indicators of resistance acquisition. From the mRNAs which we had previously validated through RT-qPCR, we selected two candidates with high TE and high protein abundance in VCaP^ER^, but also three with low TE ratios, which represent VCaP^CRPC^ cells, for further study (Figure 3B, C; Supplemental Figure S9). To validate the importance of candidate genes in the pathology of PCa in patients, we analyzed the publicly available TCGA patient database. As proteomics data is seldom available in this database, we analyzed the link between mRNA abundance of our candidates in patient samples and key indicators of PCa grade or resistance. We show that for both high TE (enriched in polysomes in VCaP^ER^) and low TE (enriched in polysomes in VCaP^CRPC^) candidates, mRNA abundance is often linked with a higher occurrence of the PCa-specific ETS fusion (79) (Supplemental Figures S11A and S12A), a higher AR score (Supplemental Figures S11B and S12B), which is linked to PCa development, and to higher detection of the AR-V7 splice variant of the androgen receptor (Supplemental Figures S11C and S12C). Only the high TE mRNA *GDAP1* was significantly and positively associated to NEPC score of patient samples, which constitutes an indicator for an advanced PCa stage, and often occurs after development of resistance (80) (Supplemental Figures S11D and S12D).

However, increased expression of a protein can occur in cancers due to several mechanisms, which include driver and passenger mutations (81), increased proliferation (82), and unstable metabolism and altered protein synthesis (83). Therefore, an increase in translation of an mRNA and accumulation of its protein does not necessarily mean that it would promote cancer resistance to therapy. Therefore, to determine if our identified candidates could play a role in the development of resistance, we performed overexpression experiments. VCaP^CRPC^ stably expressing either candidates or controls under a doxycycline-controlled TetO promoter (Supplemental Figure S13) were generated and grown with or without ENZ (Figure 3D and E). As expected, VCaP^CRPC^ and mock derivatives expressing a control protein remained sensitive to ENZ. However, expressing two candidates that have high TE ratio resulted in ENZ resistance (Figure 3F); whereas, expressing candidates with low TE ratios did not induce resistance to ENZ in VCaP^CRPC^. Taken together, these results suggest that genes encoding mRNAs that escape the general translation downregulation observed in ENZ-resistant cells are potential drivers of PCa resistance to enzalutamide.

### lncRNAs associate with ribosomes and drive ENZ resistance in VCaP^ER^

**Figure 4.**
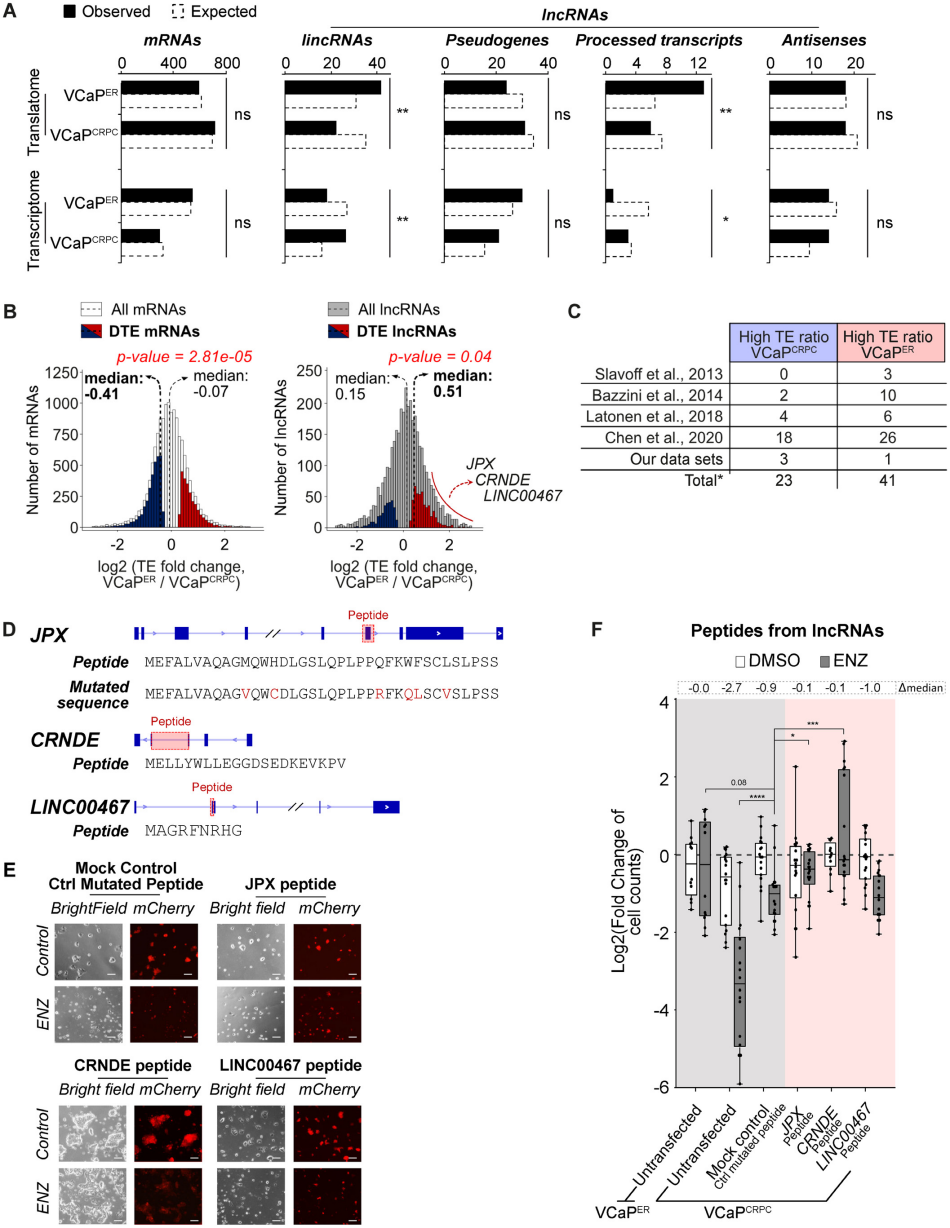
lncRNAs associate to ribosomes and drive ENZ resistance in VCaP^ER^. (**A**) Observed and expected gene counts for different categories of genes in the translatome and transcriptome RNA-seq of VCaP^ER^ compared to VCaP^CRPC^. (**B**) TE ratio distributions for mRNAs and lncRNAs. Significantly different TE ratios in VCaP^ER^ compared to VCaP^CRPC^ are highlighted (red for higher and blue for lower TE ratio). Medians are indicated with dashed lines (thin lines for all genes, bold line for genes with significant differences in TE ratio). (**C**) Table of number of lncRNAs detected as peptides in peptidomics datasets. *Total values account for lncRNAs detected in multiple studies. (**D**) Schematic of lncRNA candidates and sequences of their putative peptides. Red boxes indicate putative peptide-coding regions. (**E**) Representative pictures of VCaP^CRPC^ cell lines overexpressing putative peptide-coding sequences from candidate lncRNAs, or appropriate controls. Scale bar: 100 uM. (**F**) Fold changes of viable cell counts for cell lines overexpressing candidate lncRNAs with ENZ treatment normalized to control without ENZ treatment. Differences between ENZ-treated and control in log2(fold changes) are indicated as Δmedian. *n* = 2 or 3 biological replicates. *P-value < 0.05; **P-value < 0.01; ***P-value < 0.001; ****P-value < 0.0001. Only significant comparisons are shown.

In the past few years, several studies have highlighted association of previously unsuspected RNAs to ribosomes in the context of cancer, with wide-ranging consequences on cancer biology (84–87). For example, non-canonical association of non-coding RNAs (ncRNAs) with ribosomes has been shown to result in the production of functional peptides in multiple cancers, controlling tumor initiation or growth (88–94). Moreover, polysome profiling datasets enable us to easily investigate such unconventional RNA associations to ribosomes. We therefore sought to identify polysome-associated non-coding RNAs in our ENZ-resistance model (Figure 4A). We found that while mRNAs associate less with polysomes in resistant PCa cells, the same is not true for non-coding RNAs. Specifically, long intergenic non-coding RNAs (lincRNAs) and non-coding processed transcripts were found at a frequency much higher than expected in the VCaP^ER^ polysomal fractions (Figure 4A). Furthermore, while the global decrease in logged TE ratio previously observed (Figure 3B) was maintained in coding mRNAs (median = −0.41 for mRNAs with a differential TE) (Figure 4B, left panel), we found the opposite in long non-coding RNAs (lncRNAs), which exhibited a generally higher logged TE ratio in VCaP^ER^ (median = 0.51 for lncRNAs with a differential TE), (Figure 4B, right panel). Of note, the term ‘translation efficiency’ is not used here to imply that these lncRNAs are indeed translated, but rather as a metric for normalized association to polysomal fractions.

The above findings show that a portion of detected lncRNAs (∼13% of all detected lncRNAs) is preferentially associated with ribosomes in VCaP^ER^, potentially encoding peptides in ENZ-resistant cells. To identify if any of these ribosome-bound lncRNAs could encode peptides, we searched for putative peptides produced from lncRNAs with differential TE ratios in VCaP^ER^ and VCaP^CRPC^ in our MS data, as well as in other publicly available datasets (35,95–97), detecting 189 lncRNAs potentially encoding peptides (Supplemental Table S9). Among these, 41 originated from lncRNAs exhibiting higher TE ratios in VCaP^ER^, for example the known lncRNAs *JPX* (associated to various cancers such as lung or cervical cancer (98,99)) and *CRNDE* (associated to colorectal cancer (100,101)), while 23 lncRNAs with low TE ratio were also detected (Figure 4C). Through RT-qPCRs, we validated TE ratios in VCaP^CRPC^ and VCaP^ER^ for three high TE ratio lncRNAs (*JPX*, *CRNDE* and *LINC00467*) and one low TE ratio lncRNA (*RNASEH1-AS1*), for which peptides had been detected (Supplemental Figure S14A; Tables S3 and S4). Interestingly, we show that two of these lncRNAs, conserve the same high polysome association in the ENZ-resistant MR49F cells, when compared to ENZ-sensitive LNCaP cells (Supplemental Figure S14A). The same cannot be said however of the previously explored high-TE mRNA candidates, *GDAP1* and *OCIAD1*, which do not show higher association to polysomes in MR49F (Supplemental Figure S14B). This suggests that peptide coding lncRNAs are more prone to drive ENZ-resistance in multiple contexts of PCa.

Hence, we evaluated the potential of selected putative lncRNA-derived peptides in driving ENZ-resistance in PCa, using overexpression experiments as described above (Figure 3D; Supplemental Figure S13). Due to isoform diversity and difficulties in identifying the properly expressed transcripts of lncRNAs (102), we limited our assays to putative peptides potentially encoded from three lncRNAs. These lncRNAs, *JPX* (103,104), *CRNDE* (100,101) and *LINC00467* (105–107), were previously characterized in the context of cancer, and all exhibit high TEs in VCaP^ER^. We expressed the putative coding sequences of *JPX*, *CRNDE* and *LINC00467*, in VCaP^CRPC^ cells. We observe that overexpression of either *JPX*’s or *CRNDE*’s putative peptides induces resistance to ENZ in VCaP^CRPC^, while untransfected cells and mock cells expressing a control peptide sequence (random PCR-induced missense mutations in the JPX peptide) remain sensitive to ENZ (Figure 4D-E). To validate whether this effect is cell line-specific or could rather be applied to PCa ENZ resistance in general, we performed overexpression of putative peptides and mRNA candidates (Figure 3C) in the ENZ-sensitive LNCaP cells. Interestingly, overexpression of all of the three lncRNA-derived putative peptides decreased sensitivity to ENZ (i.e. promoted cell survival in ENZ treatment) in LNCaP cells, whereas coding genes did not (Supplemental Figure S15). This suggests that lncRNAs with a high translation potential may play an important role in the development of ENZ resistance in PCa, through their putative peptides.

### Alternative splicing of lncRNAs promotes putative coding isoforms in VCaP^ER^

**Figure 5.**
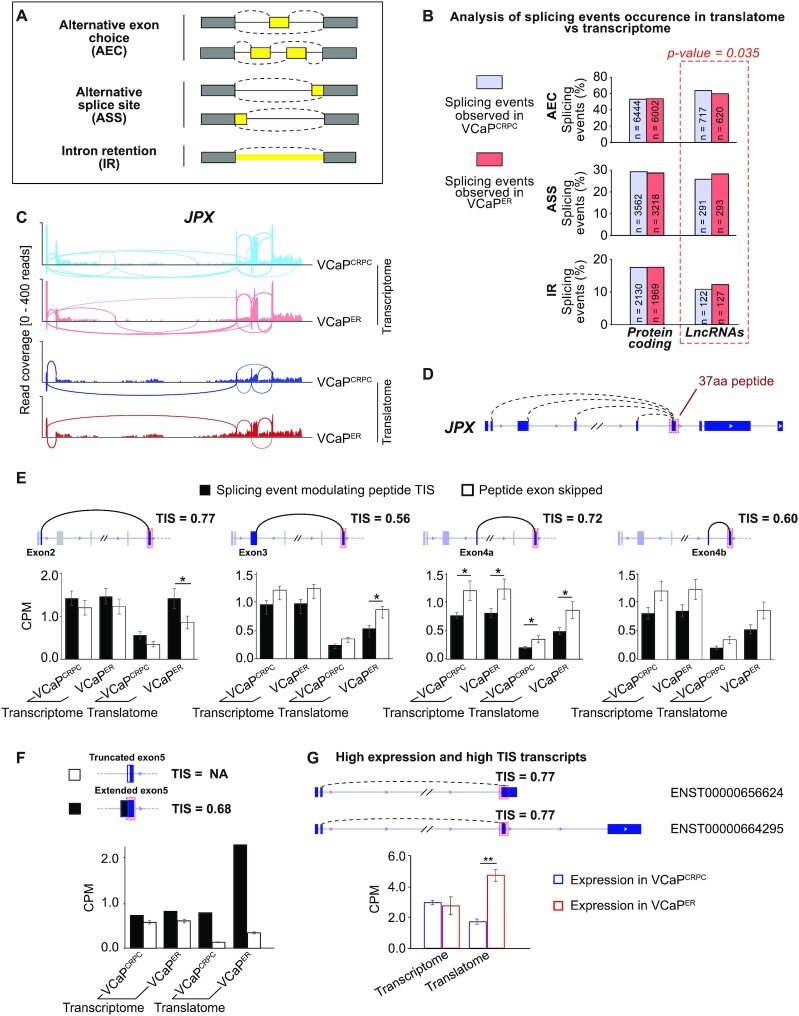
Alternative splicing of lncRNAs promotes putative coding isoforms in VCaP^ER^. (**A**) Schematic of alternative splicing event types. (**B**) Analysis of alternative splicing events with rMATS (53,54) to compare splicing events occuring in the translatome to those in the total transcriptome. Bargraphs show percentage of alternative events detected and compared through chi-squared test. Number of events are indicated. (**C**) Read density histograms of the *JPX* locus for transcriptomes and translatomes of VCaP^CRPC^ or VCaP^ER^. (**D**) Schematics of the *JPX* locus with exons (boxes) and introns (lines) indicated. Black dashed curves indicate alternative splicing events modifying the putative peptide's (exon 5) TIS. (**E**) Schematics of *JPX* exons 1 to 5 and of alternative splicing events changing the putative peptide's TIS (top). Predicted TIS scores are indicated. Quantification of corresponding transcripts in VCaP^CRPC^ and VCaP^ER^ translatomes and transcriptomes (black) and of transcripts skipping exon 5 (white) (bottom). Dark blue boxes: included exons, pastel blue: potentially included exons, grey: excluded exons. Of note, exons 4a and 4b are mutually exclusive. (**F**) Schematic of *JPX* exon 5 and quantification of transcripts with alternative splice site choice for exon 5. Extended (white) and truncated (black) exon 5 are shown with corresponding TIS scores. (**G**) Schematic of the *JPX* locus and quantification of high-TIS score (0.77) transcripts in VCaP^CRPC^ (blue) and VCaP^ER^ (red) translatomes and transcriptomes. Red boxes indicate putative peptide-coding sequences. Error bars indicate standard error of the mean, SEM. *P-value < 0.05; **P-value < 0.01.

We next investigated the mechanisms that may enable non-coding RNAs to encode peptides in ENZ-resistant cells. One such option would be alternative splicing, which causes modifications in the sequence of mature RNA molecules and has been shown to alter ribosome occupancy on RNAs (108,109), but also to control sub-cellular localization for RNAs (110,111). This is of particular interest as lncRNAs tend to be retained in the nucleus (112), whereas a cytoplasmic localization is necessary for translation. We showed that expression of some components of the spliceosome complex (i.e. SNRNP70, HSPA8, LSM3/7) was decreased in VCaP^ER^ cells (Figure 2E, Supplemental Figure S3B). This could induce changes in the splicing patterns of RNAs. To verify if alternative splicing of lncRNAs could explain their observed preferential association to ribosomes in ENZ-resistant cells, we performed a transcriptome-wide alternative splicing analysis (53,54). We searched for alternative splicing events that may occur differentially between the transcriptome and the translatome of VCaP^CRPC^ and VCaP^ER^ (Figure 5B). We found that, while mRNAs exhibited similar splicing event patterns between VCaP^CRPC^ and VCaP^ER^, lncRNAs did not (Figure 5A-B, Supplemental Table 10). We observed a significant increase in the proportion of alternative splice site choice (ASS) and intron retention (IR) for lncRNAs in the VCaP^ER^ translatome. This suggests that specific isoforms of lncRNA, but not of mRNAs, are being targeted by ribosomes in VCaP^ER^ compared to VCaP^CRPC^, and that alternative splicing may therefore be used by enzalutamide-resistant cells to promote lncRNA association to ribosomes.

We thus analyzed in more detail the splicing events occurring in the lncRNA *JPX*, as overexpression of its putative peptide induces ENZ resistance in VCaP^CRPC^ and LNCaP cells (Figure 4E, F, and Supplemental Figure S15). We wondered if alternative splicing events in *JPX* isoforms may either preferentially include the exon encoding this putative peptide or increase its coding potential. We found multiple alternative splicing events occurring in *JPX*, several of which may contribute directly to the splicing to include *JPX*’s fifth exon, host to the putative peptide (Figure 5C, D, Supplemental Table S11). For example, we observed that in the translatome of both VCaP^CRPC^ and VCaP^ER^, *JPX*’s exons 3, 4a or 4b are skipped (Figure 5C), which is not often the case in the transcriptome of these cell lines. Such exon inclusion/exclusion events can alter the sequence directly adjacent to the peptide start codon (ATG) within exon 5, the Kozak sequence, and affect translation efficiency. To investigate whether alterations in the Kozak sequence for *JPX* isoforms may affect translation efficiency of the peptide in VCaP^ER^, we performed translation initiation site (TIS) prediction using the TISpredictor software (55). Higher TIS scores are associated with higher propensity for ribosome binding and therefore increased translation potential. This analysis revealed that *JPX*’s isoform that skipped exons 3, 4a and 4b exhibits a strong Kozak (GAGAAGAUGG) and a higher TIS score (0.77) (Supplemental Figure S16). This isoform was also significantly more associated to the VCaP^ER^ polysomes than isoforms lacking exon 5 (i.e. the peptide) (Figure 5E). The inclusion of either exons 3, 4a or 4b resulted in lower TIS scores: 0.56, 0.72 and 0.60, respectively (Figure 5E, Supplemental Figure S16). However, isoforms containing any of these three exon inclusion events were less enriched in VCaP^ER^ polysomes compared to isoforms where exon 5 is skipped. Taken together, these results suggest ENZ-resistance (i.e. enrichment in VCaP^ER^ polysome) promotes selection of *JPX* transcripts containing alternative splicing that leads to increased TIS scores (0.77) of the *JPX*’s peptide. Moreover, *JPX*’s TIS score is comparable to the ones of several protein coding genes such as *AR* (0.77), the housekeeping gene *UBE2D3* (0.79), or our coding candidates with high TE ratios *GDAP1* (0.84) and *OCIAD1* (0.75). We also observed alternative splice site events occurring in *JPX*, which either extended the putative peptide-coding exon 5 in the 5’ direction or truncated it. Transcripts with truncated exon 5 lacked the peptide sequence's start codon, and correspondingly were depleted from polysomal fractions (Figure 5F). Comparatively, transcripts with an extended 5’ sequence prior to the peptide sequence displayed high presence in VCaP^ER^ polysomes, but not VCaP^CRPC^ polysomes, and a TIS score of 0.68. Finally, we found that some of the highly prevalent *JPX* transcripts also contained the peptide-coding sequence with a high TIS score (0.77) and were consequently enriched in the VCaP^ER^ translatome. Therefore, alternative splicing of the *JPX* lncRNA increases its putative coding potential, specifically in VCaP^ER^, and overexpression of a transcript containing the peptide-coding sequence with this high TIS score promotes ENZ-resistance (Figure 4D and E).

Altogether, these results show that alternative splicing of lncRNAs could lead to splice variants which may differentially bind to ribosomes, and potentially be translated into peptides that affect ENZ resistance in PCa.

## DISCUSSION

Genomic alterations in cancer are often viewed as the top of the hierarchy driving cancer biology through downstream transcription and subsequent translation to protein (113–116). While the directionality of this flow of information remains correct, the relationship between genomic, transcriptomic and proteomic levels of this hierarchy are not necessarily direct and linear. Indeed, many regulatory mechanisms are responsible for finely controlling the processes that leads to mature proteins in our cells, and several of these regulatory steps are altered in cancer. Here, we report, to the best of our knowledge, the first detailed analysis of the translatome in drug resistant PCa. For this, we generated and took advantage of novel models of ENZ-resistance which recapitulate key clinical features of the trajectory from low- to high-grade PCa, and later to antiandrogen-resistant cancer.

We found that the main pathways deregulated in ENZ-resistant PCa cells were implicated in translation and mitochondrial activity. We show increased mitochondrial content and activity in our ENZ-resistance model, which is consistent with the efficiency reported in PCa clinical trials for therapeutics targeting mitochondrial processes such as Gossypol, the G3139 anti-sense oligonucleotide and 2-deoxy-d-glucose (117–122). Our model also highlights a decrease in expression for certain ribosomal proteins, for example RPL9 and RPL29, and translation regulators such as eIF4B and 4E-BP1, previously implicated in PCa resistance in various models (70–72,123). Consequently, we show a general decrease in translation in ENZ-resistant cells, both for our novel VCaP^ER^ model, but also for the previously generated MR49F cell line. This may suggest that development of drug resistance in PCa could stem from a reprogramming of translation. Our data are consistent with studies reporting an association between cancer resistance and perturbation of translation mechanisms, through upregulation or downregulation of certain translation factors and signalling pathways such as mTOR, eIF4B or eIF2 (7,123). Recent studies have also shown that affecting these pathways and regulators of translation is a promising target for cancer therapy (7,124).

While there is an abundance of information from transcriptomic and genomic PCa analyses now accessible, detailed and specific data available on translational changes is scarce. Previous studies have demonstrated that focusing only on either genomic or transcriptomic data for instance, paints an incomplete picture of cancer, and that work to integrate multiple types of data is necessary for a better understanding of the disease (125). Here, we show that only 20% of RNAs significantly more abundant in our drug resistant PCa model are also enriched in polysomal fractions (119 out of 608 genes), indicating a low concordance between transcription and translation. Proteome and translatome data are hence essential for integrative approaches to discover novel biomarkers and therapeutic targets. We demonstrate a pipeline for discovery of such potential new targets for drug resistant PCa, through the combined transcriptomic, translatomic and proteomic data from resistance models and publicly available patient data. We discovered that several genes escape the decrease in global translation. These genes could conceivably be used in the future as markers to guide therapeutic options. Out of the identified target genes, *GDAP1* represents a highly interesting candidate, with elevated protein levels in resistant PCa and RNA expression linked to various determinants of high grade and resistant PCa. Simply overexpressing GDAP1 in the ENZ-sensitive VCaP^CRPC^ cells recapitulates a VCaP^ER^-like resistance. While this effect is not conserved when overexpressing GDAP1 in LNCaP cells, this could be due to fundamental differences between the LNCaP and VCaP cell lines (e.g. differences in the state of AR (61); mutated in LNCaP versus Wild-type in VCaP, differences in proliferation or in the transcriptional landscape). Interestingly, GDAP1 has been shown to be implicated in regulating the mitochondrial network in some contexts, with mutations in this protein affecting mitochondria volume and activity (126,127). As VCaP^ER^ show similarly increased mitochondrial activity and quantities, further research will need to assess if this role for GDAP1 is conserved in PCa, or if affecting its expression could represent a therapeutic option for combatting ENZ resistance. These early results are highly encouraging however and seem to indicate GDAP1’s value, first as a biomarker for PCa enzalutamide resistance, but also as a protein with high therapeutic potential. Additionally, it is interesting to note that, while we do show that our methodology for discovering novel resistance-associated genes in PCa discovers some interesting targets, it does not negate the need for functional validation in PCa models.

Our study also shows that translation perturbation is not limited to protein coding genes in resistant PCa, but also affects non-coding genes. Indeed, while it is well known that disruptions in the regulation of coding genes are important in cancers, recent evidence has also shown that non-coding genes can play a major role (128). We show that this also seems to be the case in PCa ENZ resistance. Indeed, we found that contrary to the global downregulation of translation in VCaP^ER^, lncRNAs tend to instead be enriched in polysomal fractions. It has previously been shown that lncRNAs may bind ribosomes in a context-specific manner (129) and some have further been shown to produce functional peptides (130,131). We demonstrate that in ENZ-resistant PCa cells, despite translation being generally down-regulated, ribosomes target some specific typically non-coding genes. However, association to ribosomes alone does not mean that translation of these lncRNAs occurs. There are many instances of lncRNAs binding ribosomes for purposes other than being translated, and even more for which ribosome-association has been detected, but their underlying functions remains to be defined (84). Hence, through analysis of several peptidomic datasets, we show that some of the identified lncRNAs can be translated into detectable peptides in some contexts and could therefore potentially encode these peptides in ENZ-resistant cells. Functional validation was performed for putative peptides from three such LncRNAs: (i) the nuclear lncRNA *JPX*, which plays roles in regulating X-chromosome inactivation in mammalian cells (103,104) and has also been shown to regulate several cancers (98,99), (ii) the generally cytoplasmic lncRNA *CRNDE*, whose role in colorectal cancer is already well established (100,101), and iii) the lncRNA *LINC00467*, whose roles in multiple cancers are now starting to be well established (105–107). Interestingly, despite their canonical non-coding functions, *JPX, CRNDE* and *LINC00467* have all already been shown to encode peptides in some contexts (89,106,132). We demonstrate that overexpression of the peptide-coding sequence of these lncRNAs is sufficient to induce ENZ resistance in the ENZ-sensitive VCaP^CRPC^ and LNCaP cell lines. This suggests a role for these putative peptides in PCa ENZ resistance and may provide a logic behind the increased binding to ribosomes of these three lncRNAs in VCaP^ER^.

We also show differences in the splicing events detected in polysome-associated RNAs compared to those found generally in the transcriptome. These differences are more prevalent in lncRNAs than in coding mRNAs, and hint at the fact that for any given lncRNA, a putative ORF may be incorporated or actively transcribed in only a subset of its isoforms. The existence of lncRNA variants could therefore explain the aberrant association to ribosomes which is observed in VCaP^ER^ and grant coding potential to otherwise non-coding genes in the context of PCa resistance (59,60). Promotion of certain splicing events in VCaP^ER^ for these lncRNAs may be due to the observed decrease in expression of key splicing regulators in VCaP^ER^, such as the U1 snRNP factor SNRNP70 or the Sm-like proteins LSM3 and 7 (77). This may in turn favor the aberrant splicing of lncRNAs into specific transcripts with high potential to be translated and would explain the increase in polysome-bound lncRNAs observed in VCaP^ER^. Several groups have highlighted changes in RNA splicing that play an important role in PCa severity or treatment resistance (133–136), but most of this work was centered around the dynamics of expression of the AR-V7 splice variant. Intriguingly, a recent study found multiple of our identified lncRNAs (such as *JPX*, *LINC00467* and *CASC2*) bound by PCa-linked alternative splicing regulators in LNCaP cells (137). While these results need to be validated in other PCa models, this mechanism, if proven true, could also contribute to the observed alternative splicing deregulation in ENZ-resistant PCa. Nonetheless, further research is needed to explore how splicing affects the translated proteins and peptides produced in ENZ-resistant cells.

Finally, while alternative splicing stands as an intriguing way for cells to regulate the coding potential of lncRNAs, other mechanisms may also be at play. Indeed, we find that the lncRNA *CRNDE* shows increased polysomal occupancy in VCaP^ER^; However, we observe no evidence of alternative splicing for this lncRNA between the two cell lines. Hence, this lncRNA may be specifically targeted by ribosomes in ENZ-resistant cells through mechanisms that still elude us thus far. Taken together, these data underscore the importance of lncRNAs as potential biomarkers and therapeutic targets in drug-resistant PCa. Here, we link, for the first time, the ribosome binding and coding potential of these lncRNAs to PCa drug resistance. Our data corroborates previous studies linking some of our candidate lncRNAs to PCa (138–140) and highlights potential novel roles for several other lncRNAs. Indeed, using lncRNAs for drug resistant PCa early detection and treatment represents an avenue of high interest due to their highly restricted spatio-temporal expression patterns and their relative ease of targeting, for example using specific anti-sense oligonucleotides (141–143).

In conclusion, our study highlights the occurrence of translation dysregulation during the development of PCa treatment resistance. We reveal an unusual shift in ribosome binding from protein coding genes to lncRNAs in ENZ-resistant PCa cells. However, while some information has been uncovered, several questions remain unclear: Is translational remodeling a consequence or driver of drug resistance? Are lncRNA-encoded peptides a prevalent occurrence in resistant PCa? Do some of the polysome-associated lncRNAs instead regulate translation of other genes, without being translated themselves? Our study advances novel concepts and prognostic biomarkers that relate to the translation output of drug resistant cancer cells and enables the discovery of potential biomarkers hidden from previous transcriptome and proteome analyses.

## DATA AVAILABILITY

All mass spectrometry files acquired in this study have been deposited to the MassIVE repository, assigned the MSV000086670 identifier and can be accessed using the ftp link: ftp://massive.ucsd.edu/MSV000086670/. Processed data for all RNA sequencing experiments are available as supplemental tables. Raw RNA-seq data was submitted to the NCBI Gene Expression Omnibus under accession number GSE179157.

## Supplementary Material

zcac034_Supplemental_Files
