# Supplementary material for: Prostate cancer resistance leads to a global deregulation of translation factors and unconventional translation
Source: NAR Cancer. 2022 Nov 4;4(4):zcac034. doi: 10.1093/narcan/zcac034 (PMC9634437; doi:10.1093/narcan/zcac034)
Supplement: zcac034_Supplemental_Files [file zcac034_supplemental_files.zip › Supplemental_figures_S1_S16.pdf]

Supplemental Figure S1

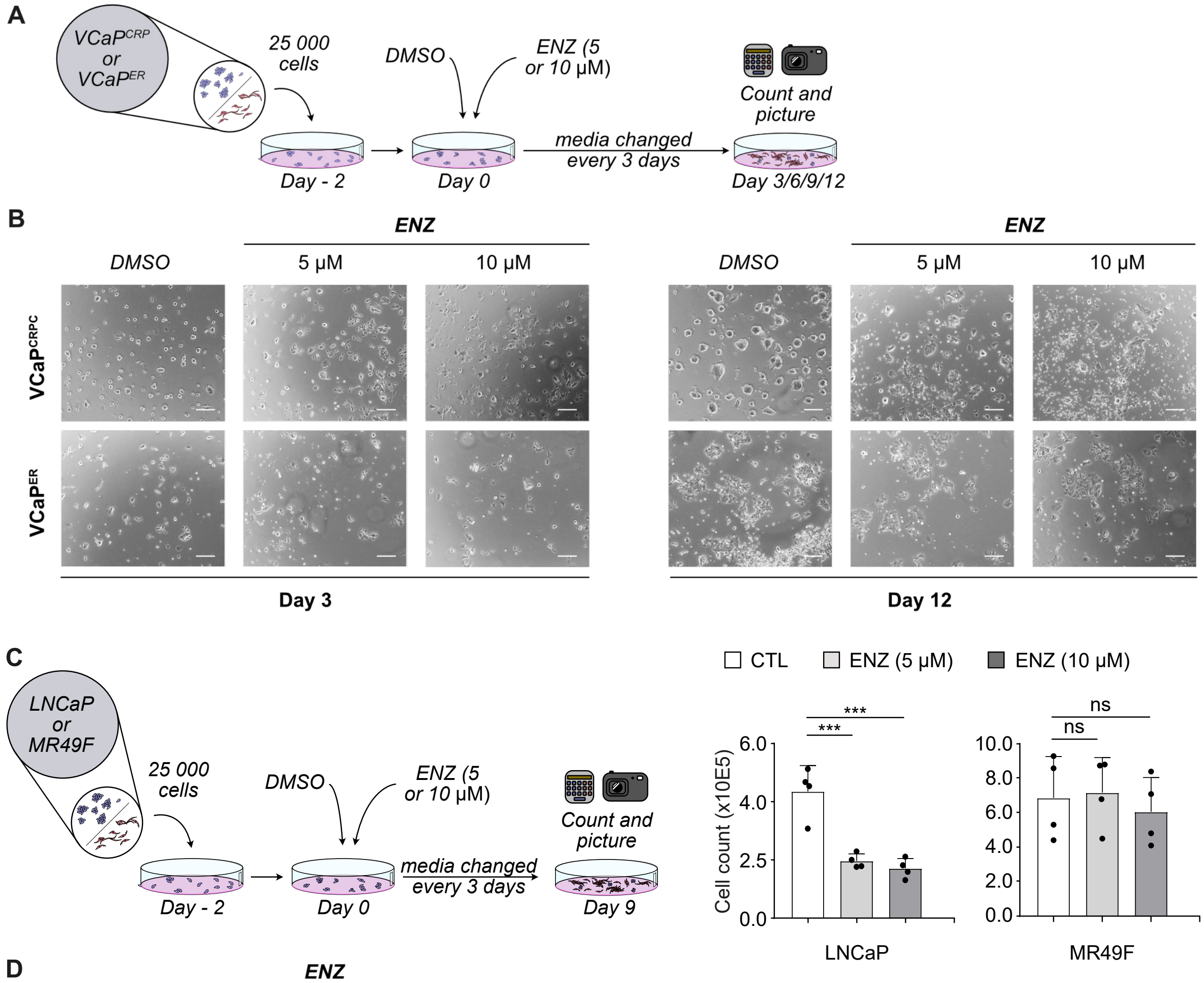

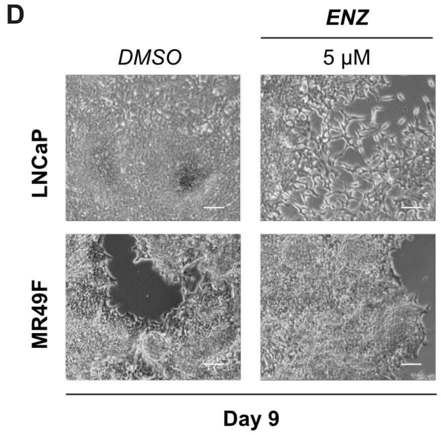

Supplemental Figure S1: Validation of enzalutamide sensitivity or resistance for PCa cell lines. (A) Schematic of cell seeding and enzalutamide treatments for VCaP^{CRPC} and VCaP^{ER}, treated with enzalutamide (0, 5 or 10 µM) for a total of 12 days, with pictures and viable cell counts taken at 3-day intervals. **(B)** Representative bright field microscopy images of VCaP^{CRPC} and VCaP^{ER} cells at indicated time points during the enzalutamide treatment. n = 3 biological replicates, with three wells per condition, counted twice each. Scale bars = 100 µm. **(C)** Schematic of cell seeding and enzalutamide treatments for LNCaP and MR49F cells, treated with enzalutamide (0, 5 or 10 µM) for a total of 9 days (left). Pictures and viable cell counts were taken at day 9, and graphed as average of cell counts for 4 biological replicates (right). **(D)** Representative bright field microscopy images of LNCaP and MR49F cells at day 9 of 5 µM enzalutamide treatment. n = 4 biological replicates, with three wells per condition, counted twice each. Scale bars = 100 µm.

Supplemental Figure S2

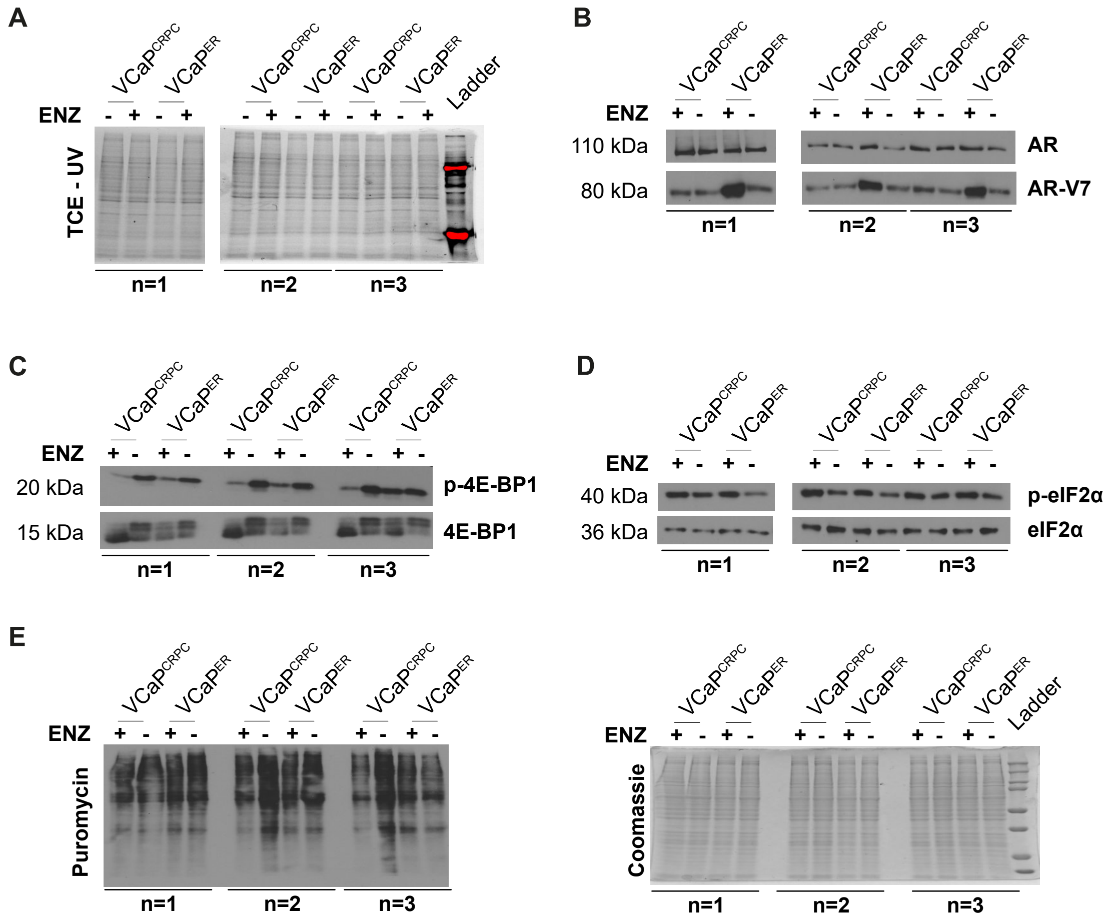

Figure S2: Full images of Western Blots and protein quantifications for AR and translation factors in VCaP^{CRPC} and VCaP^{ER}. (A) Quantification of total proteins by TCE - UV visualization, for Western Blots in B), C) and D), performed on protein extracts from three biological replicates of VCaP^{CRPC} and VCaP^{ER}, with or without 10 µM ENZ. Western Blots using **(B)** anti-AR and anti-AR-V7 antibodies, **(C)** anti-phospho-4EBP1 and anti-4E-BP1 antibodies and **(D)** anti-phospho-eIF2α and eIF2α antibodies. **(E)** Puromycylation assay in VCaP^{CRPC} and VCaP^{ER} (left) and quantification of total protein by Coomassie staining.

Supplemental Figure S3

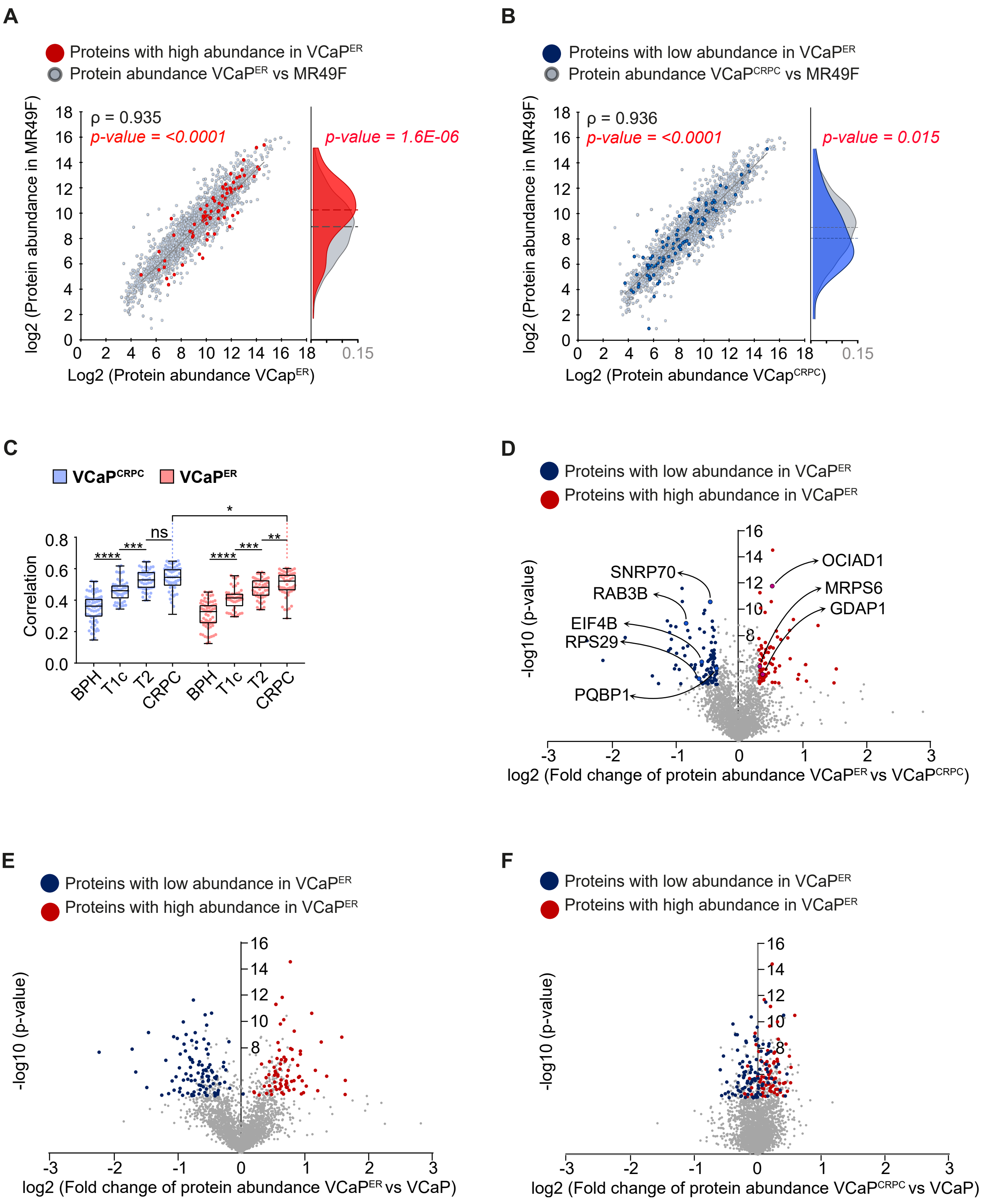

Figure S3: The VCaP^{ER} ENZ-resistance model recapitulates established features of drug-resistant PCa models. (A) Scatterplots and Spearman correlations (left) of protein abundances between ENZ-resistant MR49F and VCaP^{ER} or **(B)** VCaP^{CRPC}. Density plots (right) show MR49F expression of proteins **(A)** up or **(B)** downregulated in VCaP^{ER} compared to VCaP^{CRPC}. Grey dashed lines indicate median of the expression of MR49F total protein distribution. Colored dashed lines indicate median of the expression of proteins more (red) or less (blue) expressed in VCaP^{ER} . Significant differences between distribution was assessed by two-sample t-test. **(C)** Boxplots of correlation coefficients for VCaP^{CRPC} and VCaP^{ER} proteomes with patient proteomics samples. BPH : Benign prostate hyperplasia. T1C : Tumor not detectable by palpation but detected upon needle biopsy. T2 : Tumor detectable by palpation but confined to the prostate. CRPC : Castration resistant prostate cancer. **(D)** Volcano plots showing proteins differentially expressed between VCaP^{ER} and VCaP^{CRPC}, **(E)** VCaP^{ER} and VCaP, or **(F)** VCaP^{CRPC} and VCaP. Colored dots indicate proteins more (red) or less (blue) abundant in VCaP^{ER} compared to VCaP^{CRPC}. Proteins of interest are highlighted.

Supplemental Figure S4

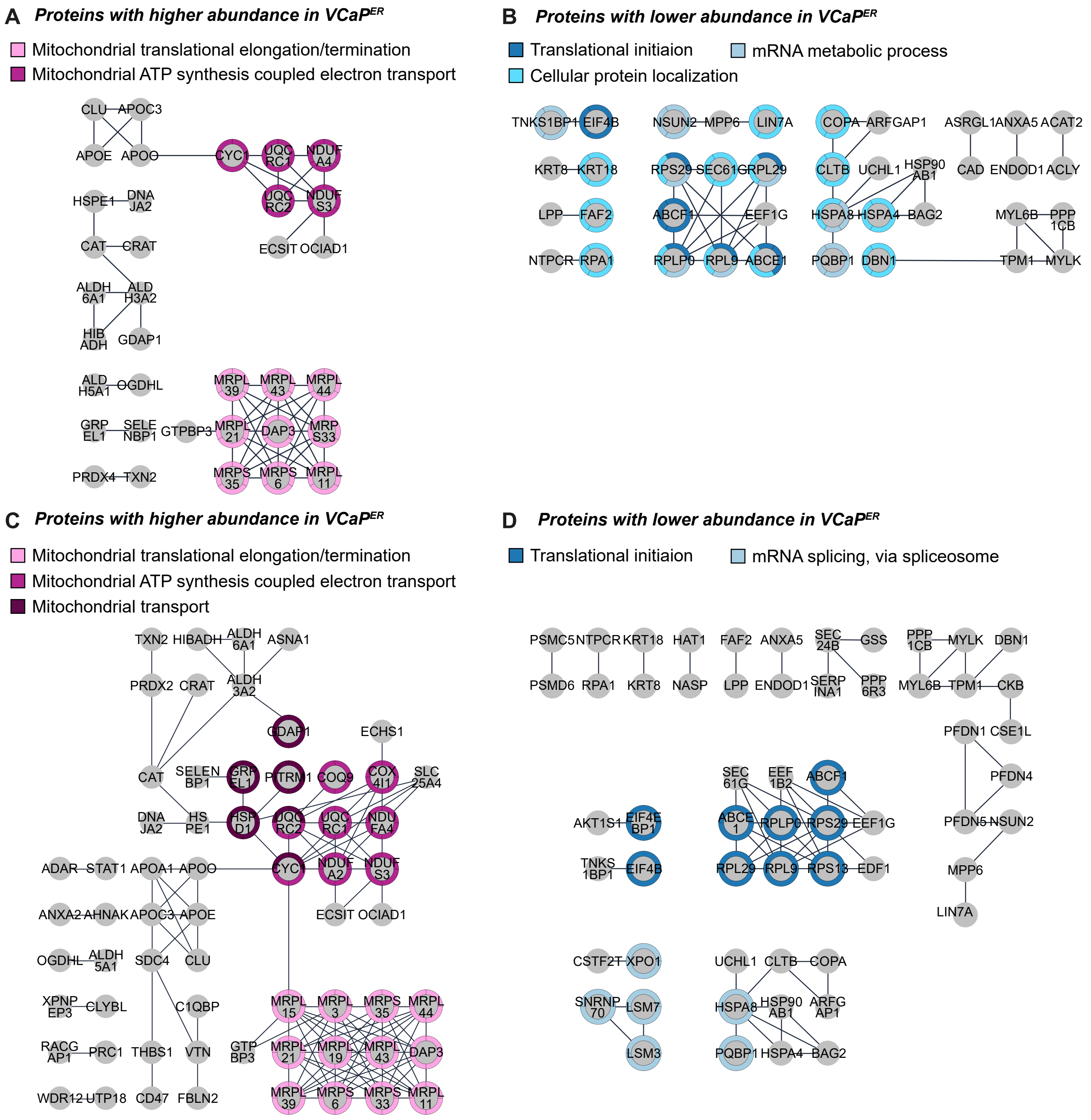

Figure S4: Proteins up- and downregulated in VCaP^{ER} assemble in protein networks. Detailed view of the network analyses of proteins differentially expressed between VCaP^{ER} and VCaP^{CRPC} presented in main Figure 2B,E. Clusters for proteins with **(A)** higher abundance in VCaP^{ER} are shown as shades of pink while those for **(B)** lower abundance proteins are shown as shades of blue. Thresholds: 0.8-1.25 fold change and **(A-B)** p-value ≤ 0.05 or **(C-D)** p-value ≤ 0.1 **(C-D)**.

Supplemental Figure S5

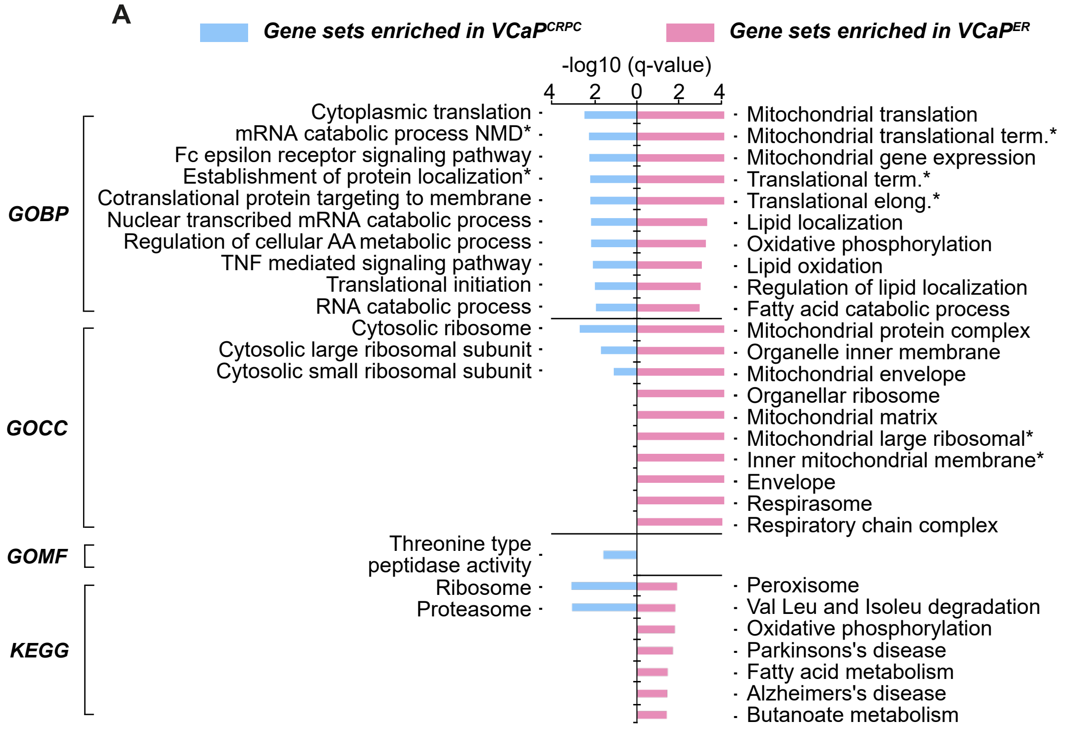

Figure S5: GSEA on VCaP^{ER} and VCaP^{CRPC} protein expression corroborates network analyses. (A) GSEA shows top 10 enriched gene sets for biological processes (GOBP), cellular components (GOCC), molecular functions (GOMF) and Kyoto Encyclopedia of Genes and Genomes (KEGG) for proteins less (blue) or more abundant (pink) in VCaP^{ER} compared to VCaP^{CRPC}. *Full names of gene sets: mitochondrial translational termination, translational termination, translational elongation, mitochondrial large ribosomal subunit, inner mitochondrial membrane protein complex, nuclear transcribed mRNA catabolic process nonsense mediated decay, establishment of protein localization to endoplasmic reticulum.

Supplemental Figure S6

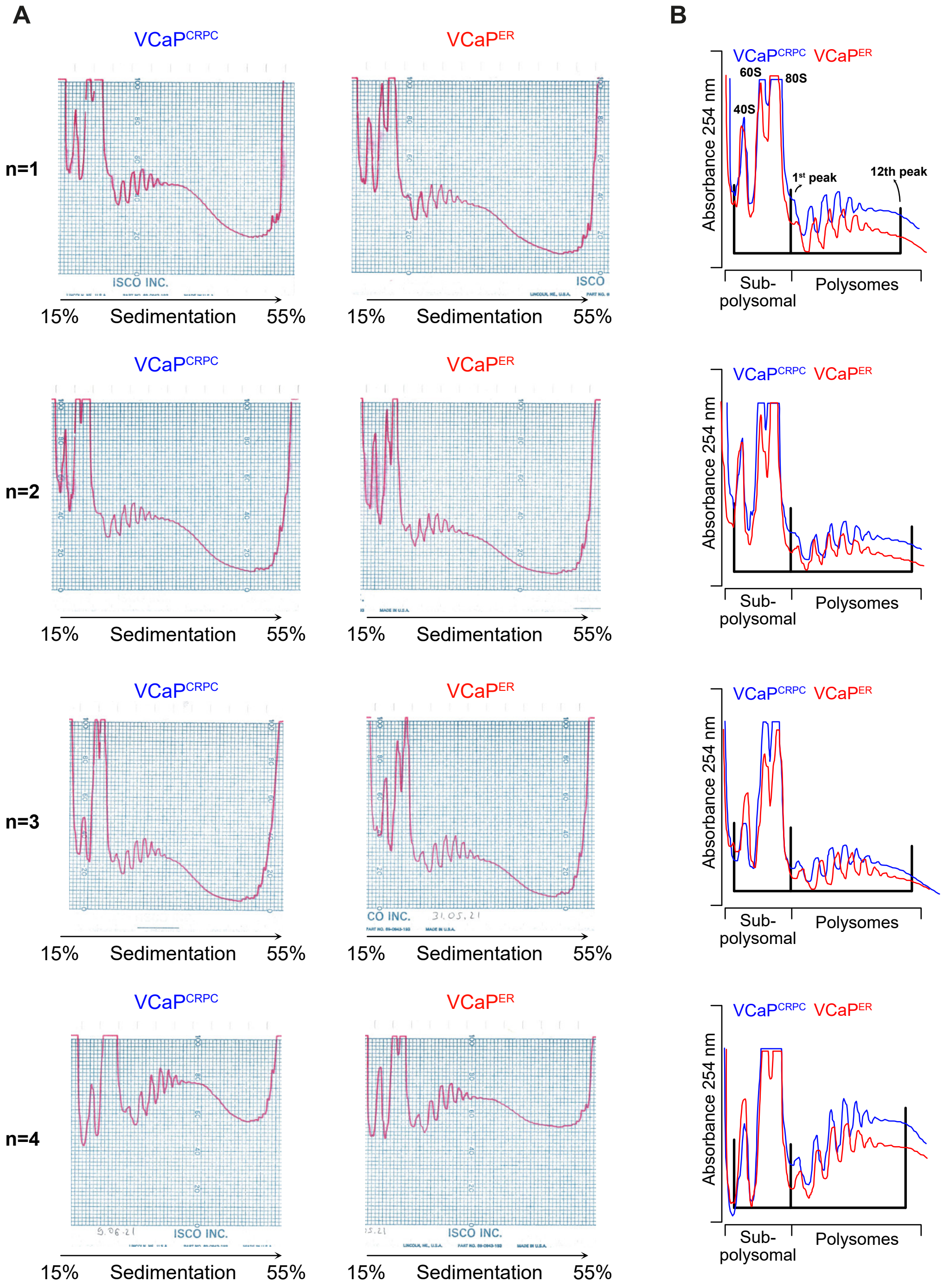

Figure S6: Polysome profiling analysis of ENZ-sensitive and -resistant cell lines. (A) Absorbance graphs and **(B)** digitized and overlayed versions for polysome profiling experiments sedimented across 15-55% sucrose gradients. Polysome profiling experiments were performed in $VCaP^{CRPC}$ (blue) and $VCaP^{ER}$ (red). Peaks show (from left to right) ribosomal subunits (40S and 60S) and mono-ribosomes (80S) (i.e. sub-polysomal fractions), light and heavy polysomes (Polysomal ribosomes), distributed along a sucrose sedimentation gradient. n=4 biological replicates.

Supplemental Figure S7

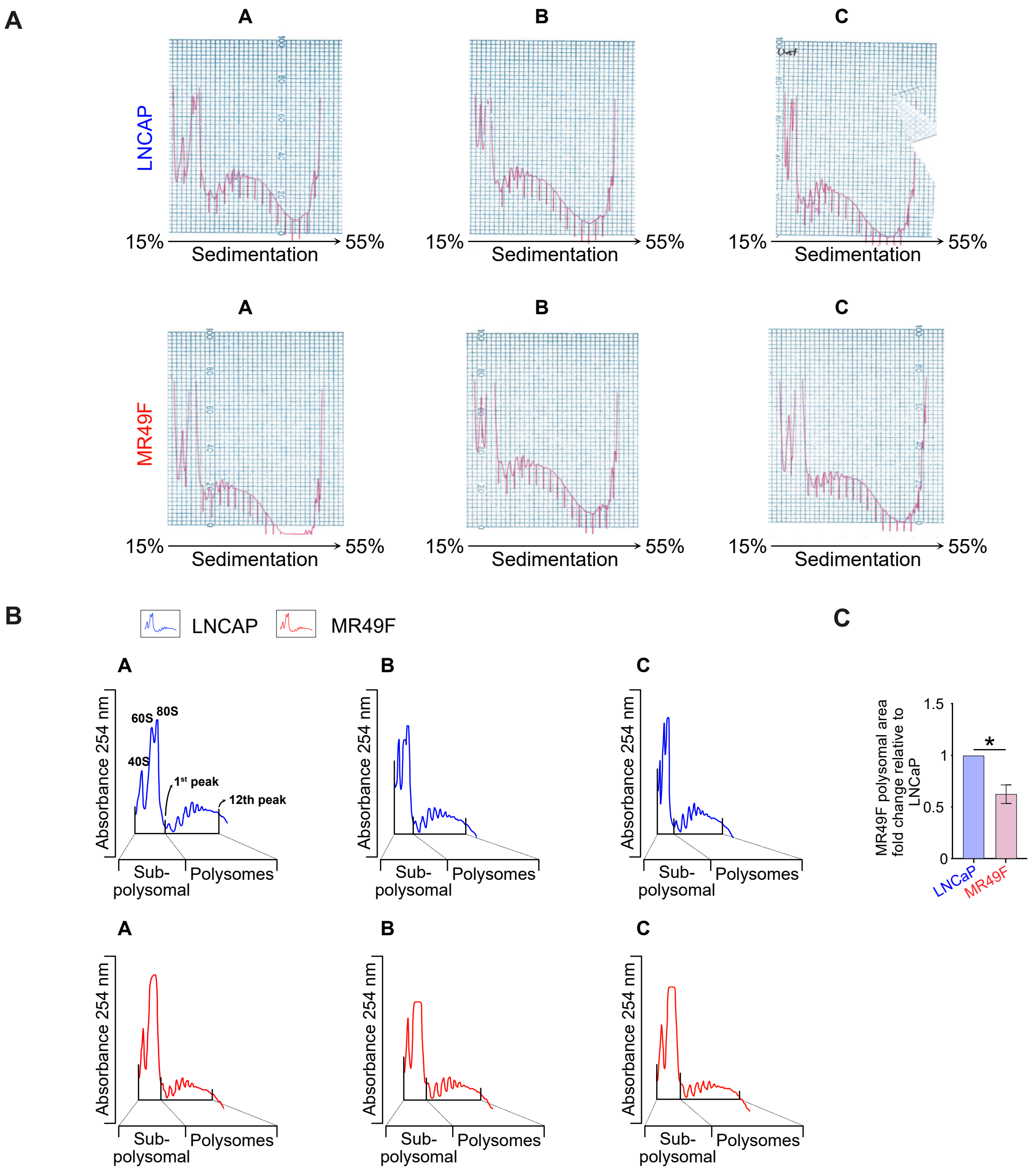

Figure S7: Polysome profiling analysis of ENZ-sensitive and -resistant cell lines. (A) Absorbance graphs and **(B)** digitized and overlayed versions for polysome profiling experiments sedimented across 15-55% sucrose gradients. Polysome profiling experiments were performed in LNCaP (blue) and MR49F (red). Peaks show (from left to right) ribosomal subunits (40S and 60S) and mono-ribosomes (80S) (i.e. sub-polysomal fractions), light and heavy polysomes (Polysomal ribosomes), distributed along a sucrose sedimentation gradient. n = 3 biological replicates (A, B and C).

Supplemental Figure S8

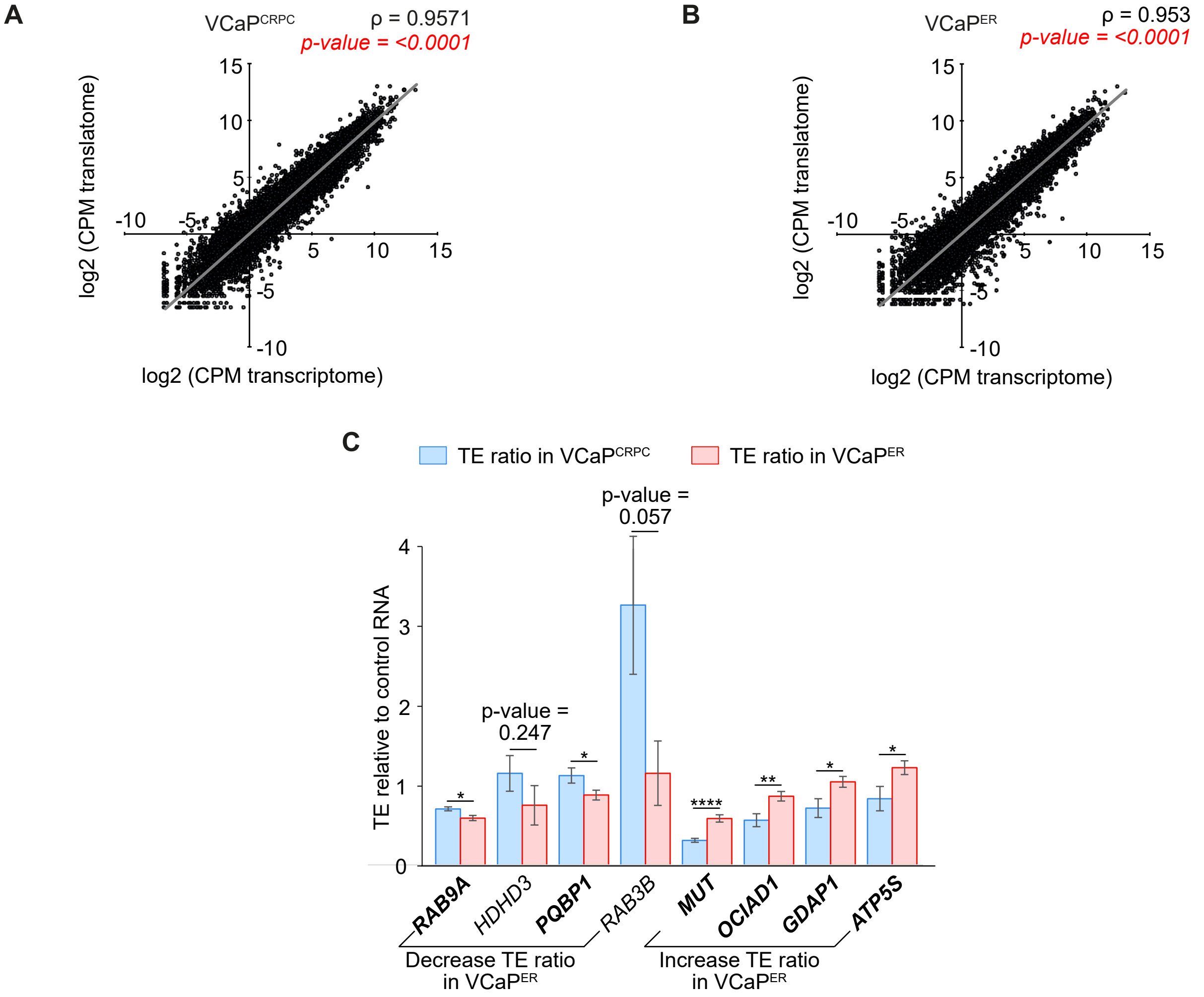

Figure S8: Analysis, validation and correlations of transcriptome and translatome RNA-sequencing datasets. **(A)** Correlation of transcriptome and translatome RNA-seq in VCaP^{CRPC} and **(B)** VCaP^{ER}. Pearson correlation coefficients (ρ) and linear regression (grey lines) are indicated. **(C)** RT-qPCR validations of TE ratio for selected candidate mRNAs in VCaP^{CRPC} and VCaP^{ER}. Candidates with similar TE ratio patterns in RT-qPCR and RNA-seq analysis are in **bold**. n=3 to 5 biological replicates. Error bars indicate standard error of the mean, SEM.

Supplemental Figure S9

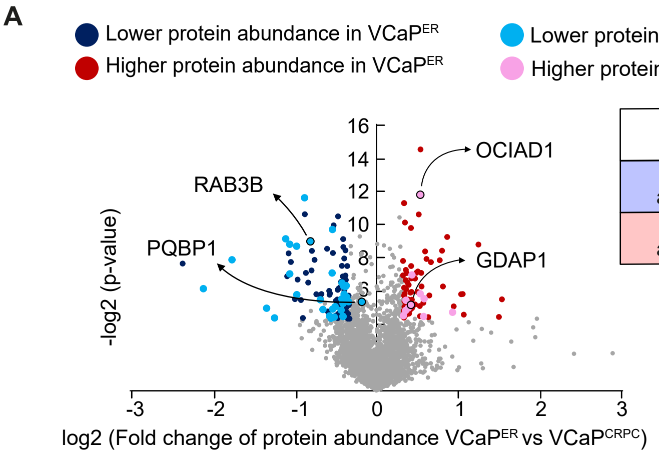

Percentage of differentially expressed proteins	
Lower protein abundance and TE ratio	30.4%
Higher protein abundance and TE ratio	12.5%

Figure S9: High TE ratio RNAs can exhibit higher protein abundance. (A) Volcano plot shows proteins differentially expressed between VCaP^{ER} and VCaP^{CRPC}. More or less abundant proteins in VCaP^{ER} compared to VCaP^{CRPC} are shown in red and blue respectively. Proteins with both a significantly upregulated TE and higher protein abundance in VCaP^{ER} are marked in pink while downregulated TE with low abundance proteins are marked in light blue.

Supplemental Figure S10

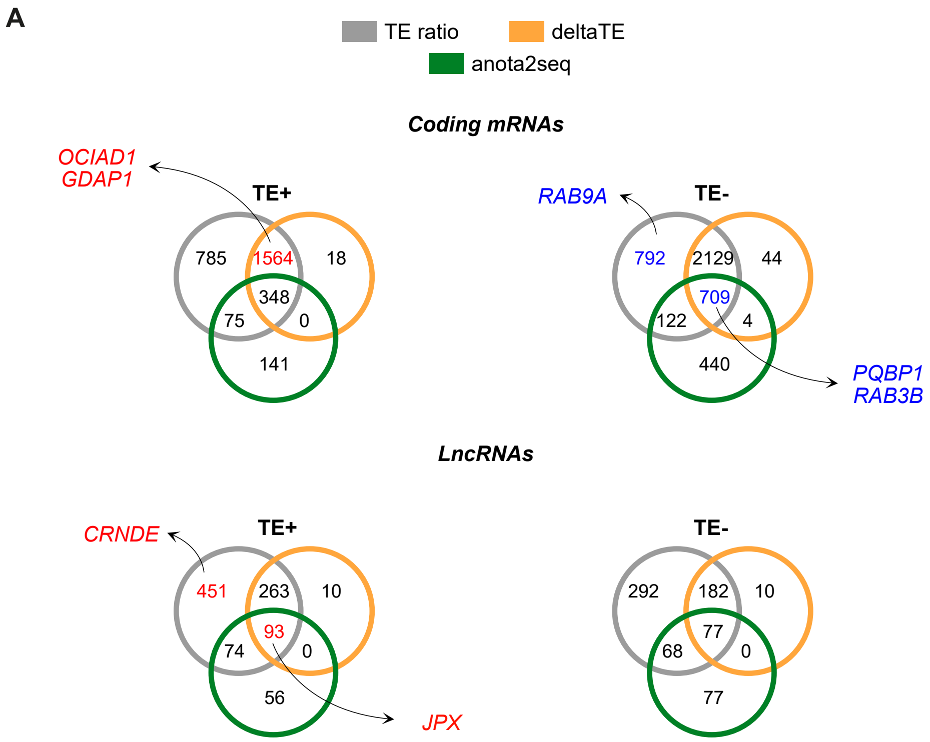

Figure S10: Validation of TE ratio calculations between VCaP^{ER} and VCaP^{CRPC}. (A) Venn diagrams showing overlap in detection of high TE and low TE mRNAs or lncRNAs from our method (TE ratio calculation) and other published methods (deltaTE (51) and anota2seq (52)). Candidate genes are highlighted: High TE ratio in red, low TE ratio in blue.

Supplemental Figure S11

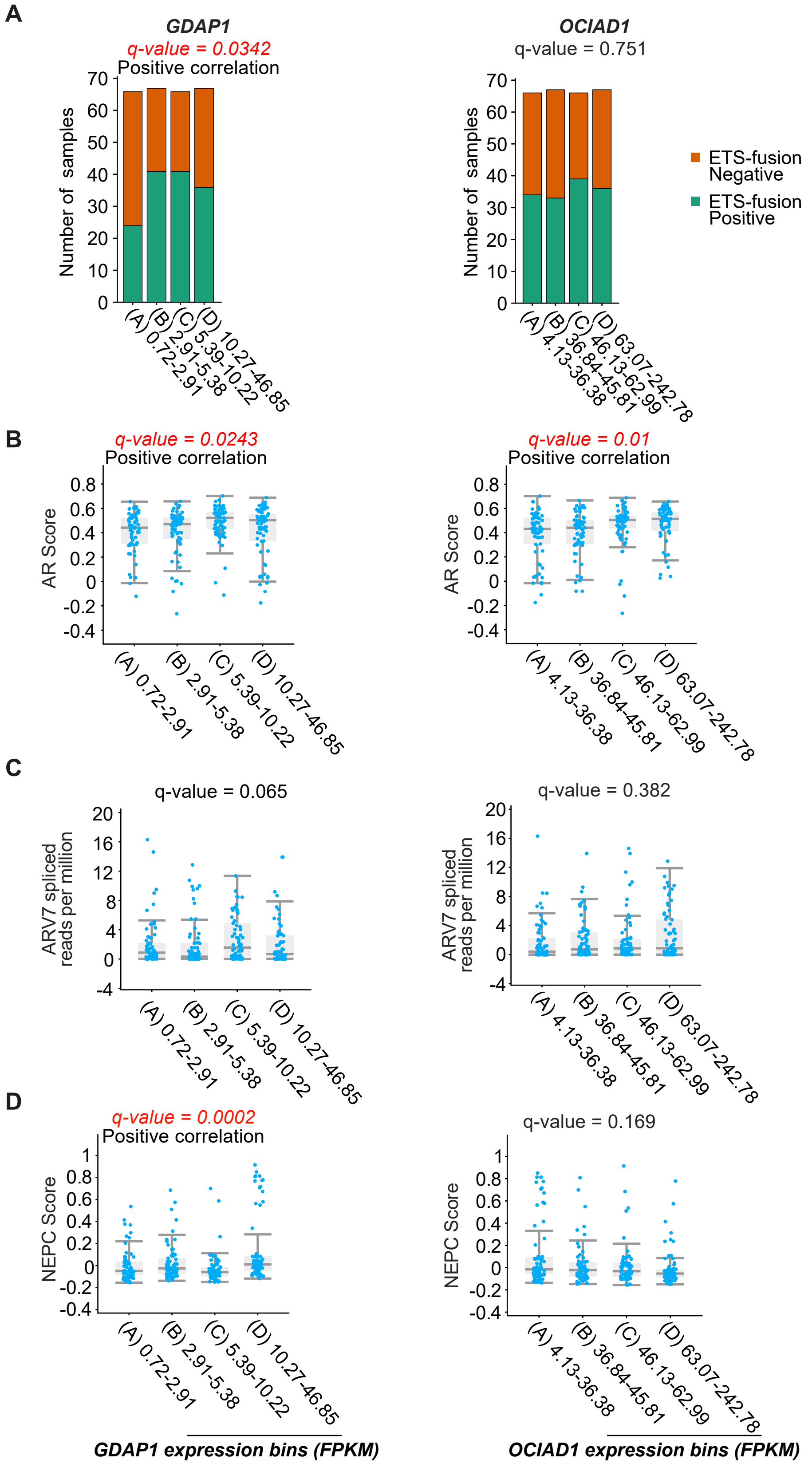

Figure S11: Expression of mRNAs with high TE and high corresponding protein abundance in VCaP^{ER} correlates with indicators of high grade and resistant PCa. Analysis of association to clinical attributes for candidate genes expression in TCGA PCa patient samples: **(A)** Occurrence of ETS fusion, **(B)** AR score, **(C)** occurence of AR splicing into the AR-V7 variant and **(D)** NEPC score, according to *GDAP1* and *OCIAD1* binned mRNA expression. Analysis was performed through cBioPortal using 36569 samples from 25 combined studies and mRNA expression (FPKM) as parameter.

Supplemental Figure S12

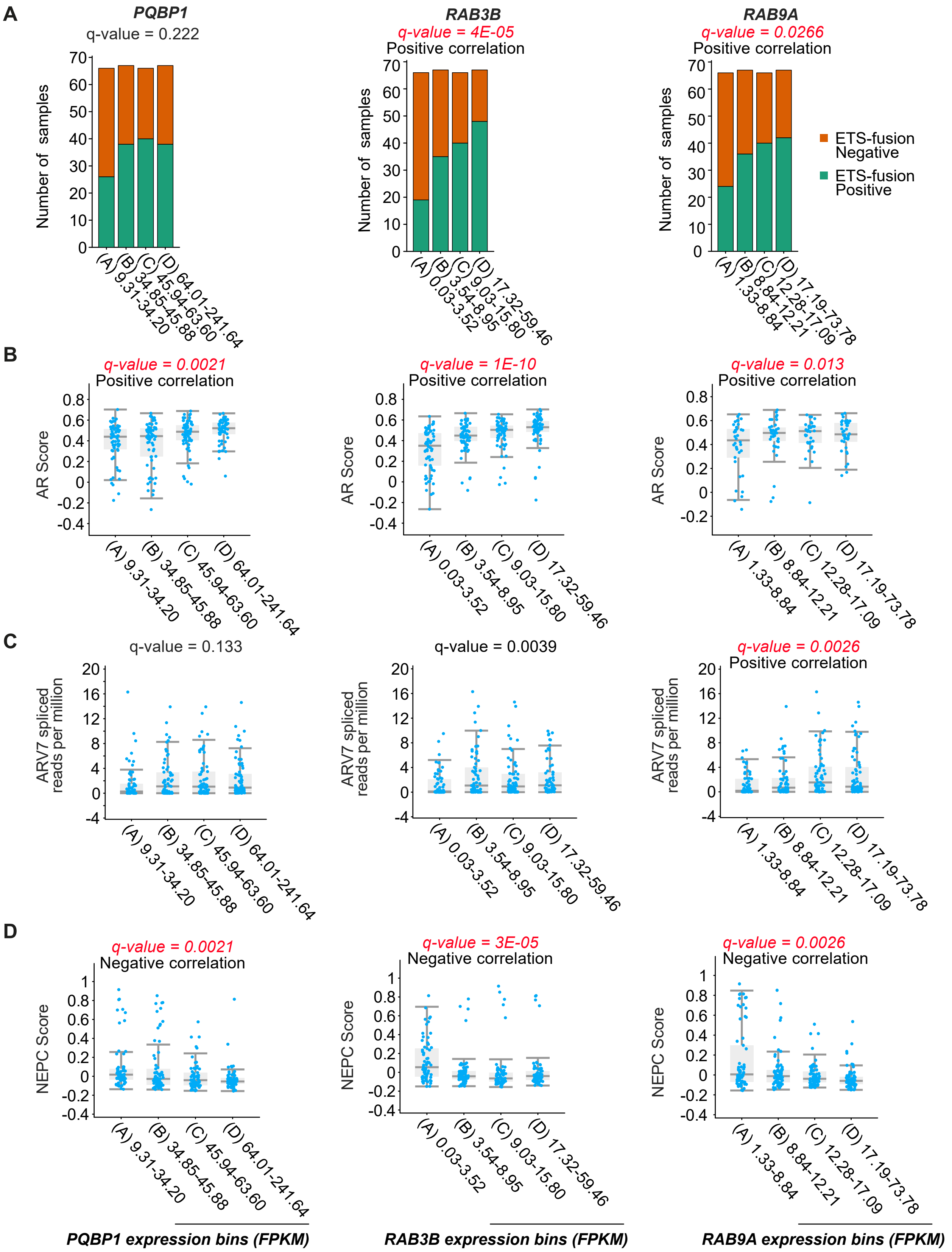

Figure S12: Expression of mRNAs with low TE and low corresponding protein abundance in VCaP^{ER} also correlates with indicators of high grade PCa. Analysis of association to clinical attributes for candidate genes expression in TCGA PCa patient samples: **(A)** Occurrence of ETS fusion, **(B)** AR score, **(C)** occurence of AR splicing into the AR-V7 variant and **(D)** NEPC score, according to *PQBP1, RAB3B* and *RAB9A* binned mRNA expression in PCa patients. Analysis was performed through cBioPortal using 36569 samples from 25 combined studies and mRNA expression (FPKM) as parameters.

Supplemental Figure S13

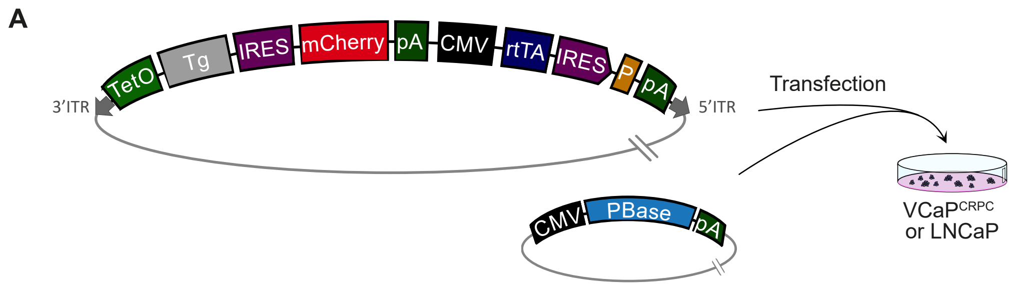

Figure S13: Overexpression stragtegy for candidate mRNAs in enzalutamide-sensitive cell lines. (A) Schematic for co-transfection of transient plasmid encoding hyPBase transposase, and vector for stable integration of a cassette containing the TetO promoter-driven mRNA of interest (transgene: Tg) with a polycistronic mCherry translated through an IRES, and a human cytomegalovirus promoter (CMV)-driven constitutive reverse tetracycline-controlled transactivator (rtTA) and puromycin-resistance gene (P). pA: poly-A signals.

Supplemental Figure S14

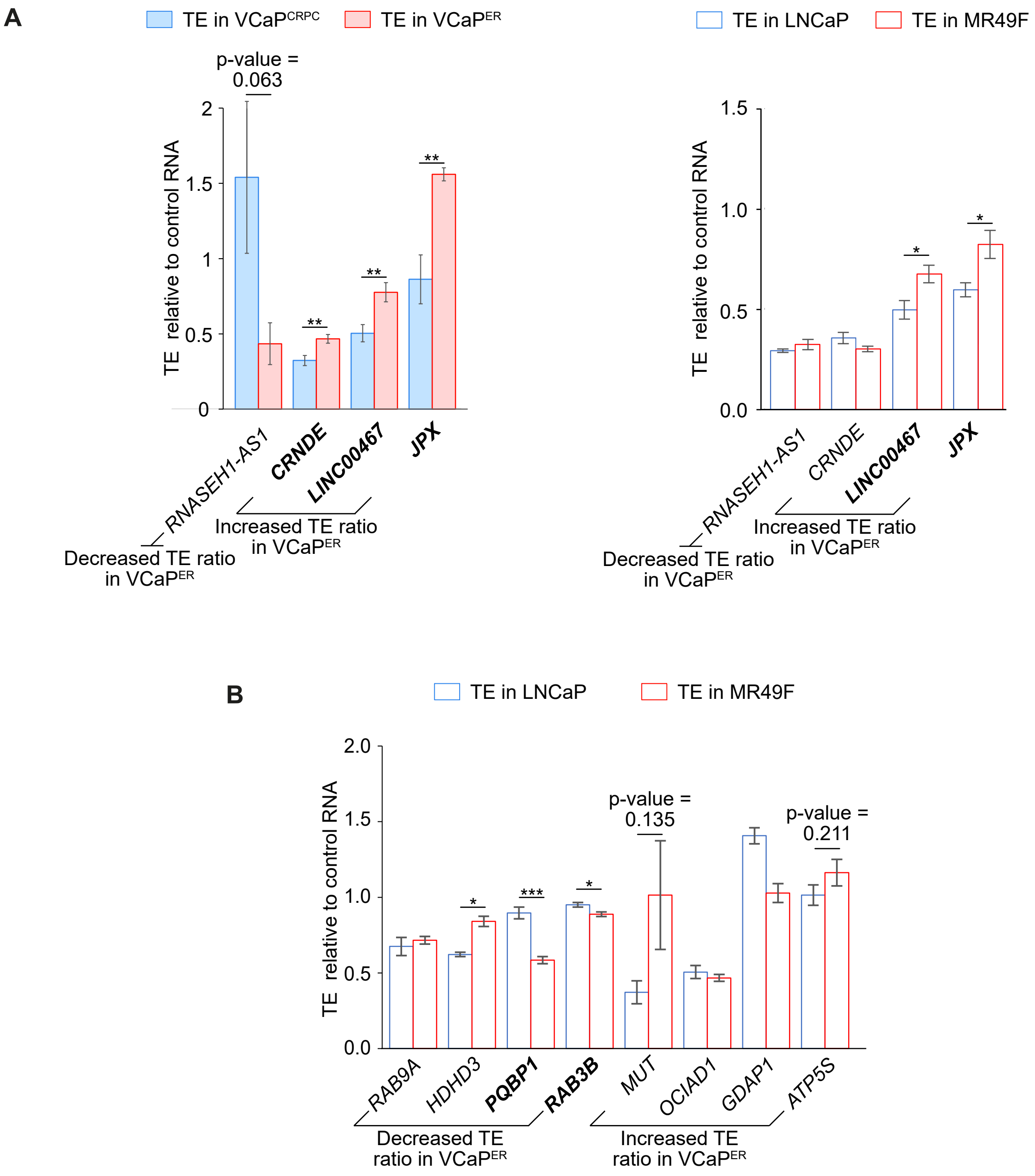

Figure S14: Validation of expression of candidate lncRNAs and mRNAs ENZ-resistant and -sensitive models. RT-qPCR validations of TE ratio for **A)** selected candidate lncRNAs in VCaP^{CRPC} and VCaP^{ER} (left) or LNCaP and MR49F (right) and **B)** selected candidate mRNAs in LNCaP and MR49F. Gene candidates with similar expression patterns in RT-qPCR and RNA-seq analysis are in **bold**. n=3 to 5 biological replicates. Error bars indicate standard error of the mean, SEM.

Supplementary Figure S15

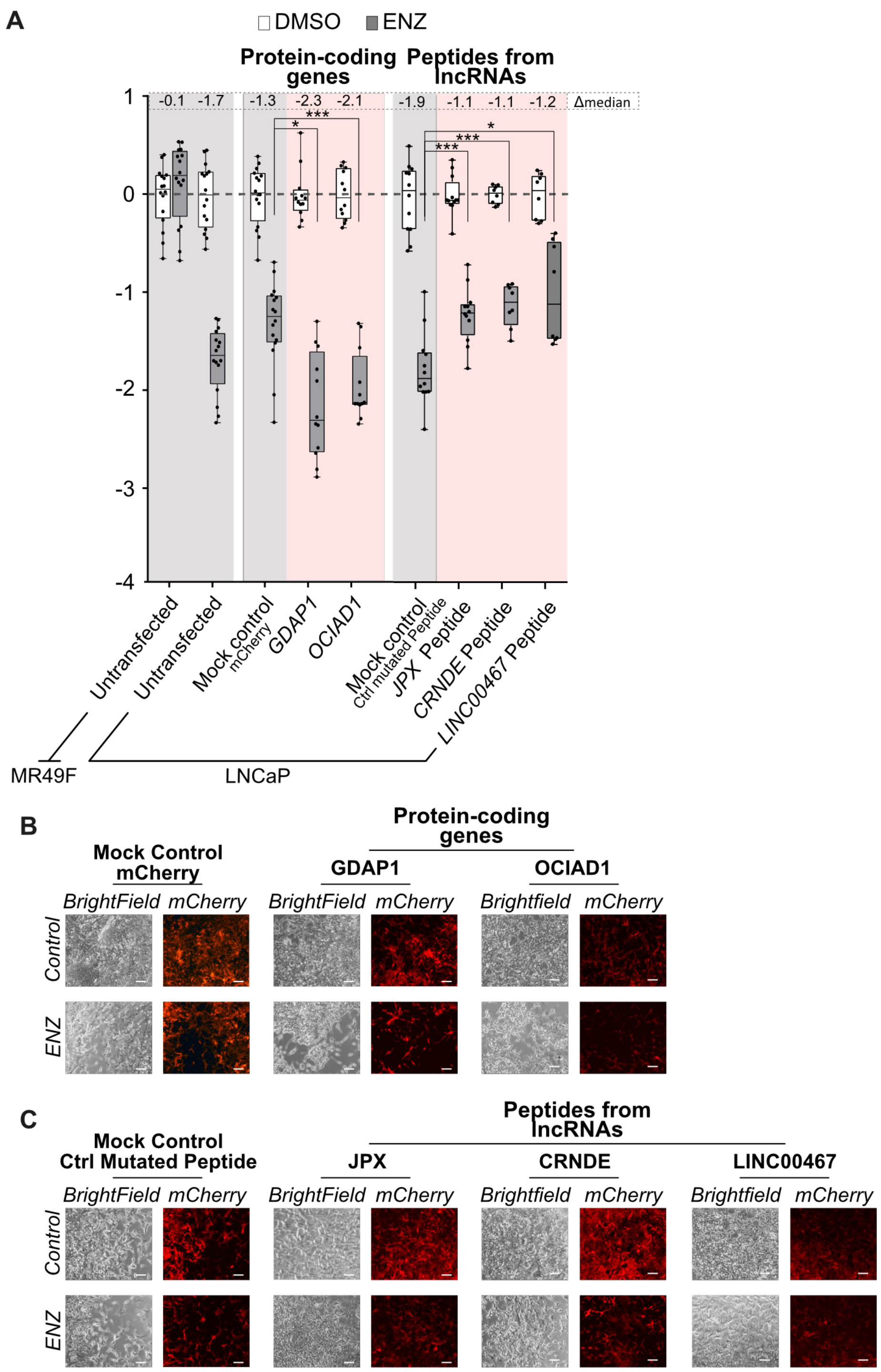

Supplemental Figure S15: Overexpression of protein or peptide candidates in LNCaP cell line. (A) Fold changes of viable cell counts for LNCaP cells overexpressing candidate genes with ENZ treatment, normalized to cells without ENZ treatment. Differences between ENZ-treated and control in log2(fold changes) are indicated as Δmedian. n = 2 or 3 biological replicates. *: p-value < 0.05; **: p-value < 0.01; ***: p-value < 0.001; ****: p-value < 0.0001. **(B)** Representative images of cell lines for GDAP1 and OCIAD1 and **(C)** JPX, CRNDE and LINC00467. Scale bar: 100 uM.

Supplemental Figure S16

A

Aternative splicing event	TIS score	TIS
Skipping of exons 3, 4a and 4b	**0.77**	GAGAAGATGG
Inclusion of exon 3	0.56	CGACTGATGG
Inclusion of exon 4a	0.72	TGTATGATGG
Inclusion of exon 4b	0.6	TTACAGATGG
Extended exon 5 in 5'	0.68	TTTGAGATGG

Figure S16: *JPX's* isoforms that contain the putative peptide sequence and that associate with ribosomes in VCaP^{ER} show high TIS score. (A) Alternative splicing events affecting TIS (55) for JPX's putative peptide. Predicted TIS score and sequence are indicated. Highest TIS score is in **bold**.
